# Electrical stimulation affects the differentiation of transplanted regionally specific human spinal neural progenitor cells (sNPCs) after chronic spinal cord injury

**DOI:** 10.1186/s13287-023-03597-w

**Published:** 2023-12-20

**Authors:** Nandadevi Patil, Olivia Korenfeld, Rachel N. Scalf, Nicolas Lavoie, Anne Huntemer-Silveira, Guebum Han, Riley Swenson, Ann M. Parr

**Affiliations:** 1 Department of Neurosurgery, Stem Cell Institute, University of Minnesota, 2‐214 MTRF, 2001 6th St. SE, Minneapolis, MN 55455 USA; 2 Massachusetts Institute of Technology, 77 Massachusetts Ave, Cambridge, MA 02139 USA; 3 Department of Mechanical Engineering, College of Science and Engineering, University of Minnesota, 1100 Mechanical Engineering Building, 111 Church St. SE, Minneapolis, MN 55455 USA; 4 Department of Neurosurgery, Stem Cell Institute, University of Minnesota, MMC 96, 420 Delaware St. SE, Minneapolis, MN 55455 USA

**Keywords:** Spinal neuronal progenitor cells, Spinal cord injury, Human induced pluripotent stem cells, Tail nerve electrical stimulation, Cell therapy

## Abstract

**Background:**

There are currently no effective clinical therapies to ameliorate the loss of function that occurs after spinal cord injury. Electrical stimulation of the rat spinal cord through the rat tail has previously been described by our laboratory. We propose combinatorial treatment with human induced pluripotent stem cell-derived spinal neural progenitor cells (sNPCs) along with tail nerve electrical stimulation (TANES). The purpose of this study was to examine the influence of TANES on the differentiation of sNPCs with the hypothesis that the addition of TANES would affect incorporation of sNPCs into the injured spinal cord, which is our ultimate goal.

**Methods:**

Chronically injured athymic nude rats were allocated to one of three treatment groups: injury only, sNPC only, or sNPC + TANES. Rats were sacrificed at 16 weeks post-transplantation, and tissue was processed and analyzed utilizing standard histological and tissue clearing techniques. Functional testing was performed. All quantitative data were presented as mean ± standard error of the mean. Statistics were conducted using GraphPad Prism.

**Results:**

We found that sNPCs were multi-potent and retained the ability to differentiate into mainly neurons or oligodendrocytes after this transplantation paradigm. The addition of TANES resulted in more transplanted cells differentiating into oligodendrocytes compared with no TANES treatment, and more myelin was found. TANES not only promoted significantly higher numbers of sNPCs migrating away from the site of injection but also influenced long-distance axonal/dendritic projections especially in the rostral direction. Further, we observed localization of synaptophysin on SC121-positive cells, suggesting integration with host or surrounding neurons, and this finding was enhanced when TANES was applied. Also, rats that were transplanted with sNPCs in combination with TANES resulted in an increase in serotonergic fibers in the lumbar region. This suggests that TANES contributes to integration of sNPCs, as well as activity-dependent oligodendrocyte and myelin remodeling of the chronically injured spinal cord.

**Conclusions:**

Together, the data suggest that the added electrical stimulation promoted cellular integration and influenced the fate of human induced pluripotent stem cell-derived sNPCs transplanted into the injured spinal cord.

**Supplementary Information:**

The online version contains supplementary material available at 10.1186/s13287-023-03597-w.

## Background

The estimated number of people with spinal cord injury (SCI) living in the USA is approximately 294,000, with a range from 250,000 to 368,000 persons [1]. Chronic SCI is a devastating condition that has seen few significant positive treatments in terms of functional recovery to date. It is now largely accepted that there will not be one “cure” for SCI, but rather a toolbox of potential therapies depending on the injury, that may be used alone or in combination.

Electrical stimulation is currently the most effective therapy available in humans, although mechanisms of action are poorly understood, and few animal models are available. We have developed a non-invasive method of tail nerve stimulation that we have shown to result in functional recovery after a clinically relevant contusion injury in a rat model [2]. Interestingly, in our human clinical studies, we have also observed that some patients develop long-term neuroplastic changes resulting in restoration of volitional movement even after the stimulator is turned off [3]. These neuroplastic changes remain unexplained.

The transplantation of human induced pluripotent stem cells (iPSCs) has also been a potential proposed therapy, especially when regionally specified to the spinal cord [4]. It has been suggested, however, that without any type of “training” of new neurons, the appropriate connections may not result, and this may explain why even transplanted neurons that appear to integrate into the injured spinal cord do not result in robust functional recovery [5]. Therefore, here we propose combining our unique sNPCs with tail nerve electrical stimulation (TANES). Human sNPCs, developed in our laboratory [6], are a type of stem cell that is regionally specific to the human spinal cord. These cells are generated from iPSCs and are designed to be autologous [7]^.^so that future human clinical trials will not require immunosuppression as with embryonic or fetal derived neural stem cells. There has been renewed interest in neural stem cells (NSCs) after recent publications have demonstrated that they can integrate into the injured spinal cord and produce functional benefit in both rodent and primate models [8–11]. However, some previous studies of neural stem cell transplantation have not produced functional benefit, and there are likely several reasons for this. One reason is that the cells have been brain-derived and not spinal-derived neurons, and there is now evidence that this does in fact play an important role [9]. Thus, regional specificity [12] is key in a successful transplantation paradigm [13].

It has been suggested that the formation of a glial scar affects axonal growth, regeneration [14–16], and functional recovery and our initial hypothesis also included scar ablation in our chronic model in addition to cell transplantation and TANES. We have successfully ablated the scar without damaging spared spinal cord tissue utilizing a rose Bengal based phototoxic approach [17, 18]. Hence, we first proposed to utilize this method in combination with cell transplantation, potentially providing a more permissive environment for cell survival, integration, and differentiation. The results of this first set of experiments laid the foundation for the second set of experiments, determining whether or not glial scar ablation therapy should be included in the second set of combinatorial experiments.

In the second set of experiments, we proposed to examine the influence of TANES on the differentiation of sNPCs and to further examine integration between the new neurons and the host spinal cord. Therefore, we first transplanted human iPSC-derived sNPCs in combination with glial scar ablation to determine whether our glial scar ablation method would improve sNPC integration into the injured chronic rat spinal cord. The results informed the second study, which determined the effects of transplanted sNPCs after chronic SCI when utilized in combination with TANES.

## Methods

### Overview of the project

The adult female athymic nude (ATN) rat model was considered for chronic contusion SCI. The contusion model of spinal cord injury is the most relevant form of SCI in humans. Female rats were preferred because their bladders must be expressed in the acute period after injury and male rats are more difficult to express and develop massive hematuria in our previous experience. We utilized ATN rats because they are athymic and T cell deficient and thus do not require immune suppression for human cell transplant survival. These rats are standardized at weights of 230–250 g (adult weight) and therefore are approximately 10 weeks of age at the time of injury. All the Hsd:Athymic Nude-Foxn1nu rats were supplied by Envigo CRS (USA) Ltd. We minimized the number of rats needed for our experiments to identify statistical significance. Sample size was estimated based on our experience [17, 18] and power analysis as per Charan and Kantharia, 2013 [19]. All experiments were designed and reported in accordance with the Animal Research: Reporting of In Vivo Experiments (ARRIVE) guidelines.

In the first aim of this project, we combined scar ablation with sNPC transplantation to determine whether scar ablation would be included in the second aim. This part of the study the estimated sample size of 20 rats was considered. However, the sample size was adjusted (*n* = 24) for expected attrition (20%) (Additional file 1: Table S1). On arrival all the rats were simultaneously randomized to the treatment groups. Rats were assigned a group designation and weighed. Each rat was assigned a temporary random number within the weight range group. For each group, a cage was selected randomly from the pool of all cages. All rats received a moderate contusive thoracic injury (Infinite Horizons Impactor), and treatment was deferred for 56 days to create a chronic injury. The rats received either saline or glial scar ablation (GSA) with rose Bengal (RB) (*n* = 12 rats per group). Seven days later all rats received injections of sNPCs and were sacrificed by transcardial perfusion under isoflurane anesthesia at two time points: 8 and 16 weeks (w) after cell transplantation. Group 1: sNPC only (A and B); Group 2: GSA + sNPC (C and D). The tissue was harvested, processed, and analyzed for cavitation analysis, transplanted cell survival and differentiation pattern of the transplanted cells utilizing standard histological and immunohistochemical (IHC) techniques. Five rats were assigned for tissue analysis. The individual rat was considered as an experimental unit.

In the second aim, we combined sNPC transplantation with TANES with the estimated number of rats equals 24 to identify statistical significance. The power analysis was calculated utilizing the effect size (1.0) for Basso, Beattie, Bresnahan (BBB) locomotor rating scale with a 90% power in injured rats. Also, the numbers were adjusted for expected attrition or death of 30% (Additional file 1: Table S1). Therefore, the total number of rats required for this part of the study was 36. A total of 36 ATN rats received a chronic injury as in the first aim. On arrival, all the rats were simultaneously randomized as described in the first aim to one of the three groups (*n* = 12 rats per group). Group 1 received culture medium only (injury only); Group 2 received sNPCs (sNPC only); Group 3 received sNPCs supplemented with TANES (sNPC + TANES). Rats were sacrificed by transcardial perfusion under isoflurane anesthesia at 16 weeks post-transplantation, and tissue (*n* = 5 rats/group) was processed and analyzed for cavitation analysis, transplanted cell survival, differentiation pattern of the transplanted cells utilizing standard histological and IHC techniques. Tissue clearing techniques were performed (*n* = 1 rat/group). Functional testing was performed (*n* = 9 rats/group). The individual rat was considered as an experimental unit. In this study we were specifically interested in the influence of TANES on the differentiation pattern of the transplanted cells in the chronic SCI model of rats, and the effects on integration. Therefore, we excluded TANES-only treatment on injured rats in this current study.

All the experiments were conducted in a blinded fashion. The first investigator (NP) was the only person aware of the treatment group allocation. A second investigator (OC and RNS) was responsible for conducting TANES, functional outcomes, whereas a third investigator (AHS, GH, RS) performed data collection and tissue analysis. Finally, a fourth investigator (AMP) (also unaware of treatment) assessed, analyzed, and interpreted overall data.

### Human iPSC-derived sNPC culture

Human iPSC-derived sNPCs were cultured according to our previously published protocol [6, 7]. Human iPSCs were maintained in adherent culture at 37 °C in 5% CO2 on human vitronectin (rhVTN, AF-140-09; PeproTech, Rocky Hill, NJ) in Essential 8 Media (A-2858501; Thermo Fisher Scientific, Waltham, MA) and passaged using hypertonic citrate and the derivation of sNPCs. Briefly, cells were passaged at half the density required for maintenance culture (1:6 spit ratio) and cultured for 18–24 h before the Essential 8 media was replaced by Essential 6 media (A1516401; Thermo Fisher Scientific) supplemented with 250 nM LDN-193189 (S7507; Selleckchem, Houston, TX). Cells were then maintained under these conditions with daily media changes until passage at a 1:10 split ratio onto VTN after day 3. Following passage, the adherent cells were cultured in Essential 6 media supplemented with 250 nM LDN-193189, 100 nM Retinoic acid (RA, R2625; Sigma-Aldrich, Billerica, MA) and 3 µM CHIR99021 (4423; Bio-Techne, Minneapolis, MN) for 8 additional days. The medium was changed daily. By day 11, the cells detached as spherical cell aggregates and were collected and resuspended into Dulbecco's modified Eagle's medium (DMEM) F/12 basal (11,039–047; Thermo Fisher Scientific), containing 1 × N2 (A13707-01; Thermo Fisher Scientific), 1 × B27 (17,504–044; Thermo Fisher Scientific), 100 nM RA and 1 µM smoothened agonist (SAG, 11,914; CaymanChem, Ann Arbor, MI). Cell spheres were placed into suspension culture in ultralow attachment plates (3471; Thermo Fisher Scientific) for an additional 6 days with media changes every other day before cryopreservation. For cryopreservation, spheres were resuspended in day 11–17 media with 10% DMSO and cooled in a Mr. Frosty™ Freezing Container (5100–0001, Thermo Scientific™) overnight at − 80 °C before transferring into liquid nitrogen. The day prior to transplantation, spheres were thawed in day 11–17 media in ultralow attachment plates. On the day of transplantation, sNPCs were dissociated with TrypLE, washed and resuspended at a concentration of 50,000 cells/µL. Cells were kept on ice prior to transplantation. Regionally specific sNPCs that were defined as mixture of immature caudal and ventral neurons and restricted progenitors based on HOX gene expression, ventral domain markers, motor neuron-related transcripts, and general pathways affiliated with stemness [13] were generated using modifications to our previously described accelerated defined neural induction protocol.

### Immunocytochemistry

Immunocytochemistry (ICC) was performed. Cultures were washed twice with blocking solution and incubated 1 h with secondary antibodies. 4′, 6-diamidino-2-phenylindole dilactate (DAPI, ThermoFisher D3571) was added for 10 min before washing three times in Dulbecco's phosphate-buffered saline (DBPS). Antibodies used included: anti-GFAP (glial fibrillary acid protein, Z0334; 1:500; Dako) for astrocytes, anti-APC (adenomatous polyposis coli, ab15270; 1:100; Abcam) for mature oligodendrocytes, anti-MBP (Myelin Basic Protein, AB40390 1:250; Abcam) was used to label terminally mature, myelin-producing oligodendrocytes, βIII-tubulin (neuronal marker ab18207; 1:250; Abcam) and anti-NF200 (neurofilament protein, ab8135; 1:250; Abcam) for neurons. Sections were incubated with secondary antibody conjugated to Alexa Fluor 488 donkey anti-mouse (1:1000, ThermoFisher Scientific A-21202), Alexa Fluor 555 donkey anti-rabbit (1:500, ThermoFisher Scientific A-31572). Negative controls included unstained cultures, cultures stained with secondary only antibodies, and positively stained cultures known not to express the antigens of interest. Positive controls were also included.

### Contusion injury

Adult female ATN rats were used for this study. The study was performed in strict accordance with the recommendations in the Guide for the Care and Use of Laboratory Animals of the National Institutes of Health (NIH) (Additional file 2: Animal Handling and Monitoring). The protocol was approved by the Institutional Animal Care and Use Committee at the University of Minnesota (Protocol Number 1810-36461A). Laminectomy was performed at the T8/T9 vertebral level. Adult rats were placed in an induction box until sufficiently anaesthetized with isoflurane (1–5% inhalation with oxygen) and supplemented as needed during the surgery. The surgical site is then shaved outside the surgical area. Rats were then transferred to the sterile operating table in a prone position on a heating pad to maintain body temperature at 37 °C and a nose cone with a small hole for the rat’s nose was placed over the rat’s nose. Sterile lubrication gel was applied into the rat’s eyes to prevent corneal burns from the isoflurane. 1–5 ml/kg of sterile saline was injected subcutaneously pre-op for hydration. The surgical site was prepped with betadine and draped in a sterile fashion. An incision was made in a longitudinal direction along the direction of the spine in the approximate area of T8/9 using anatomic landmarks. The skin and subcutaneous tissue was opened until the spinous processes and adjacent paraspinal muscles are identified. Hemostasis was obtained using sterile cotton tip applicators. The T8/9 lamina are identified and removed. Once the spinal cord is sufficiently exposed a moderate contusion injury was performed with a 200Kdyn force using the Infinite Horizon Spinal Cord Impactor (IH 0400) (Precision System and Instrumentation LLC, Fairfax Station, VA). The muscles and subcutaneous layers are then re-approximated and closed in layers. Rats were injected subcutaneously with extended-release buprenorphine (1.0–1.2 mg/kg) 2-4 h before the surgical procedure. In addition, rats were injected subcutaneously with ceftiofur (1–20 mg/kg) for a period of 5 days after surgery to prevent infection. Rats were monitored daily post-surgery for signs of infection. Potential problems related to surgery include loss of bladder function, infection, weight loss, autophagia, skin breakdown. Bladders are expressed twice daily until bladder recovery; the urine was examined for gross hematuria. If any signs of bladder infection were observed, rats were treated according to accepted protocols. Athymic nude rats occasionally get fur in their eyes and require a saline flush to remove it. Rat eyes were checked and cleaned weekly. We excluded the rats that were inconsistently injured (> 210kdyn).

### Scar ablation technique

In the first study cohort, ten rats received glial scar ablation. The photoablation procedure utilizing RB is described by Zhang et al. [20, 21] and has previously been reported by our group [17, 18]. Briefly, 1 μL of 2% (diluted in 0.9% saline) RB was injected into the cavity at the injury site through a 26-gauge blunt Hamilton metal needle connected to a Hamilton syringe. This injection was made at a depth of 0.8–1.0 mm for 1 min. Eight minutes after injection, the spinal cord was illuminated for 5 min with the full-spectrum light of a halogen bulb (150W, 7 cm distance). To prevent damage by the heating source from the halogen light, the spinal cord was bathed with saline during the illuminating time. Immediately after illumination, the wound was sutured. The sNPC-only group (*n* = 12 rats) was injected with saline instead of RB.

### Cell transplantation

Laminectomy site at T8/T9 was reopened in chronically injured rats and these rats were either injected with culture media (10 μl) or sNPCs (10 μl, 500,000 cells). Cells were transplanted in 3 equal aliquots at 3 separate sites at the midline of the spinal cord, rostral, caudal, and at the lesion site. Chronically injured ATN rats were anaesthetized by inhalation of isoflurane, and the laminectomy site was reopened. A 10 µl Hamilton syringe with a 32‐gauge needle (0.5inch long, 30° beveled tip) was used to inject culture media (10 μl) or sNPCs (10 μl, 50,000cells/µl, total = 500,000 cells) divided into three separate and equal injection sites at the epicenter and 1 mm rostral and caudal to the epicenter of the lesion. Culture media or the cells were injected at a rate of 1 μl/min using a microinjector (Stoelting, Wood Dale, IL, USA). The injection needle was left in situ for five minutes after injection to minimize cellular regurgitation. The surgeon was not blinded to group allocation, but subsequent assessments were performed by blinded examiners.

### Tail nerve electrical stimulation (TANES)

TANES was started one week after cell transplantation treatment using a physical therapy instrument Pens Electrostimulator 12c. Pro, Pentheon Research, California, USA. TANES was performed using modifications to a previously described protocol by Zhang et al. [2, 21]. Electro-acupuncture stimulator, designed specifically for the clinical requirements performing percutaneous electrical needle stimulation. The strength of stimulation can be adjusted according to the response of rats to the stimulation. The rats were confined in a plexiglass cylindrical container without anesthesia such that they remained in a recumbent position during TANES treatment. The rat's tail was then connected to the instrument with two electrodes clipped to copper adjustable rings. These rings were placed adequately apart (to avoid a short circuit) on the base of the tail. When the stimulation current entered the rat's tail, the two hind limbs started to move extensively and alternatively. The rats were able to stand up, step and even freely walk during the stimulation. The strength of stimulation was adjusted to 1–4 mA at a frequency of 2–4 kHz to induce a slight vibration of the tail or twitch of the hind limbs for 10 min per session, 5 sessions a week, for a total of 16 weeks. All of the rats at one point did not move during TANES. They were all similar in their amount of movement. The rats that did not receive TANES (sNPC-only group) did undergo equivalent handling such that they were free moving when placed in a plexiglass cylindrical container without anesthesia.

### Functional studies

Locomotor activity was evaluated using the BBB locomotor rating scale [22] by two independent blinded examiners. Motor subscores were determined according to the method of Lankhorst and colleagues [23]. This test was performed before and after the initial injury, and weekly after cell transplantation and TANES treatment until sacrifice. Mechanical allodynia was tested using a modified protocol of Kostich et al. [24]. Von Frey test was performed on all the rats (Additional file 2: Supplementary Methods).

### Tissue harvesting

Rats were fully anesthetized with intraperitoneal injection of ketamine hydrochloride and transcardially perfused with 4% paraformaldehyde in 0.1 M phosphate-buffered saline (PBS), pH 7.4. Spinal cords were removed and post-fixed overnight in the same fixative solution, then immersed in sucrose (30% *w*/*v*) and washed with PBS. A segment of the spinal cord 1.3 cm in length encompassing the injury site at thoracic segment and lumbar segment (L1–L3) was removed and embedded in Tissue-Tek OCT embedding compound (VWR, Mississauga, ON, Canada). The tissue (T8–T9; L1–L3) was cryosectioned in the sagittal plane at 20 μm intervals. To analyze the percentage of proliferating endogenous NPCs and percentage of transplanted cell migration toward T7 and T10, spinal cords were sectioned in the transverse plane at 20 μm intervals using a Leica CM3050 S cryostat and mounted on positively charged slides.

### Cavitation analysis

Cavitation analysis was performed using five rats per group. Tissue was harvested in the standard fashion. Every eighth parasagittal section was processed for hematoxylin and eosin (H and E) and luxol fast blue (LFB) staining. Fifteen sections per rat (rostro-caudally from the epicenter of the cavity) were imaged on a Leica DMi8 inverted microscope. To compare the cavity area between groups, a modified protocol that combines H and E (background) and LFB (myelin) staining was developed. The area of maximum cavitation (epicenter) of each section was traced using ImageJ software from Fiji (v.1.45) (NIH, Bethesda, MD). The measurements obtained were used to generate values for the cavity volume for each of the cords from each treatment group. The total cavity volume was expressed in cubic millimeter (mm^3^).

### Immunohistochemistry

Immunohistochemistry (IHC) analysis was performed (*n* = 5 rats per group). Every eighth parasagittal section was processed for fluorescence IHC, antibodies were utilized to identify the transplanted cells with either HNA (human nuclear antigen, MAB1281; 1:250; Millipore, Billerica, MA) or SC121 (human cytoplasmic marker, AB-121-U-050; 1:250; SC Proven, Newark, CA). Differentiation was examined with dual-label immunofluorescence combining either HNA or SC121 co-localized with other IHC antibodies used in separate experiments that includes anti-Ki67 (ab15580; 1:250; Abcam) for dividing cells, anti-Nestin (ABD69; 1:250; Millipore) for neural stem cells, anti-GFAP (glial fibrillary acid protein, Z0334; 1:500; Dako) for astrocytes, anti-NF200 (neurofilament protein, ab8135; 1:250; Abcam) for intermediate filaments that are found in neurons, anti-synaptophysin (1:250, Synaptic Systems). Anti-GAT1 (GABA transporter 1, AB1570; Millipore) for neurotransmitter transporting involved in the termination of GABA transmission, and anti-VGlut1 (135316; 1:500; Synaptic Systems) for glutamate transporter to the membrane of synaptic vesicles. Also, differentiation was studied with other oligodendrocyte markers—that is, late markers anti-APC (adenomatous polyposis coli protein, ab15270; 1:100; Abcam) and anti-MBP (myelin basic protein, ab40390; 1:250; Abcam) to label terminally mature, myelin-producing oligodendrocytes. Appositions between axons and transplant-derived oligodendrocytes were visualized with the combination of antibodies against the neurofilament (NF200) and the SC121 epitope as generic axonal and transplant-derived cell markers, respectively [25]. To quantify the expression of serotonergic axons at lumber region of the spinal cord anti-5HT (5-hydroxytryptophan, 1: 75,000, ImmunoStar) was utilized. Sections were then incubated with secondary antibodies conjugated to Alexa Fluor 488, 555 or 647(A-31572, A-21206, A-21432, A-11055, A-31570 and A-21202; Thermo Fisher Scientific). Negative controls were obtained by omission of the primary antibody. Positive controls were also included. DAPI (Thermo Fisher Scientific) was used to counterstain DNA. The proportion of transplanted cells that were double labeled was calculated for each rat spinal cord examined.

### Tissue clearing and immunostaining

CLARITY [26] tissue clearing was performed on rat samples in the sNPC-only and sNPC + TANES groups. Tissue clearing was performed with an X-CLARITY system (Logos Biosystems, Annandale, VA) on rat samples in the sNPC-only (*n* = 1) and sNPC + TANES groups (*n* = 1) and tissue imaged at 20 × 0.95 NA with a ribbon confocal microscope (RS-G4, Caliber I.D., Andover, MA). For intact spinal cord tissue that underwent clearing, clearing solution was circulated continuously at 37 °C, 1.5A current, and 30 rpm pump speed until tissue cleared. After samples underwent active electrophoretic clearing, they were subjected to fluorescence immunostaining with SC121 primary antibody and Alexa Fluor 488 secondary labeling before imaging [13]. Tissue clearing technique enables large-scale volumetric imaging within three dimensional (3D) structures that allows visualization of opaque whole spinal cord tissues. Though it is a powerful tool for 3D structure and quantification, there are certain disadvantages that could lead to discrepancies in the results. The technique leads to morphological deformation of the sample and affects fluorescence signals. While the method is compatible with immunostaining, the combination of long clearing times, added to the long incubations required for standard antibody penetration techniques, may set to imperfect antibody penetration. The preliminary data with this new technique lack sufficient quantification to draw any significant conclusions; however, this was performed to demonstrate the possibility that axonal/dendritic extension occurs from the grafts using a representative sample per treatment.

### Quantification

To quantify the number of surviving transplanted cells around the injury site in the spinal cord, 15 sections per rat were obtained in the parasagittal plane at 20 μm thickness, 160 μm apart, and all cells in the 15 sections were counted. Transplanted cells expressing HNA, with typical cell morphology and clearly delineated cell borders, were counted using ImageJ software from Fiji (v.1.45) (NIH; Bethesda, MD). The cell counts were then adjusted using the Abercrombie method [27]. This number was expressed as a percentage of cells surviving with the number of live cells injected as the denominator (500,000 cells per rat) in rats that were sacrificed 16 weeks after transplantation. To analyze the migration of transplanted cells toward T7 (0.6 cm rostral to the lesion site) and T10 (0.6 cm caudal to the lesion site), three sections from both thoracic regions were cut from each rat spinal cord in the transverse plane at 20 μm thickness and the percent of absolute cell migration in the three sections was counted in reference to DAPI and averaged for each thoracic region. To analyze endogenous NSCs, three transverse sections of 20 μm thickness from T7 (0.5 cm rostral to the injection site) and T10 (0.5 cm caudal to the injection site) were considered. The percentage of Nestin-positive cells that were co-localized with Ki67 around the central canal was quantified.

The quantification of the immunofluorescent signal for GFAP (representing glial scar surrounding the cavity) was conducted using three longitudinal sections of 20 μm centered at the middle of the cord and separated by 160 μm, and results were averaged per rat. Images of these sections were acquired on a fluorescent microscope (Leica DMi8 inverted microscope) at 10 × objective. Three random fields of view per section within an area of 0.3 mm^2^ around the cavity were considered for quantification. Immunoreactivity was quantified with the ImageJ software from Fiji (v.1.45) (NIH; Bethesda, MD) by measuring the integrated optical density (intensity of fluorescence per unit of surface area) [28]. Biological variation in relation to any differences in signal absorption of any individual section was accounted for by defining the threshold. To define the threshold (T), the number (*n* = 10) of known negative and positive regions was selected randomly from three different sections from all three treatment samples. Mean intensities (u1 and u2) and standard deviations (*σ*1 and *σ*2) were obtained for negative and positive regions. The threshold was calculated as T = *u*1 * *σ*2 + *u*2 * *σ*1/*σ*1 + *σ*2 for the GFAP antibody. The average integrated density of antibody signal was recorded for each section around the lesion cavity, averaged for each rat, and compared across treatment groups. The results were expressed in arbitrary units represented as astrocyte density [17]. Equal amounts of tissue were imaged at each section from each group.

The percentage of transplanted surviving (HNA^+^/SC121^+^) cells that co-expressed/differentiated into Ki67, Nestin, GFAP, APC, NF200, MBP, synaptophysin, GAT1, or VGlut1 was quantified for the transplanted rats. The HNA/SC121-positive cells and those double labeled for the various markers were counted in three longitudinal sections of 20 μm, centered at the middle of the cord and separated by 160 μm. In each section, three images (total of nine images/rat and five rats/group) containing HNA/SC121-expressing cells were obtained at a 20 × objective Z stack using the Leica DMi8 inverted microscope. The results were averaged per rat and compared across the treatment groups [29]. To quantify the density of 5HT fibers, images were obtained from 5 or 6 parasagittal consecutive spinal cord sections from lumbar region (L1–L3). The results were averaged per rat and compared across the treatment groups.

### Data analysis

All quantitative data are presented as mean ± standard error of the mean (SEM). Statistics were conducted using GraphPad Prism. Two-tailed unpaired Student t tests were used to compare two group data, and one-way analysis of variance (ANOVA) with Bonferroni post-test was used to compare three group data (*p < 0.05, **p < 0.01; ns, non-significance). For the BBB scores, repeated-measures ANOVA was used. Significance was set at *p* < 0.05.

## Results

### Human sNPCs express markers of neuronal differentiation in vitro

**Fig. 1:**
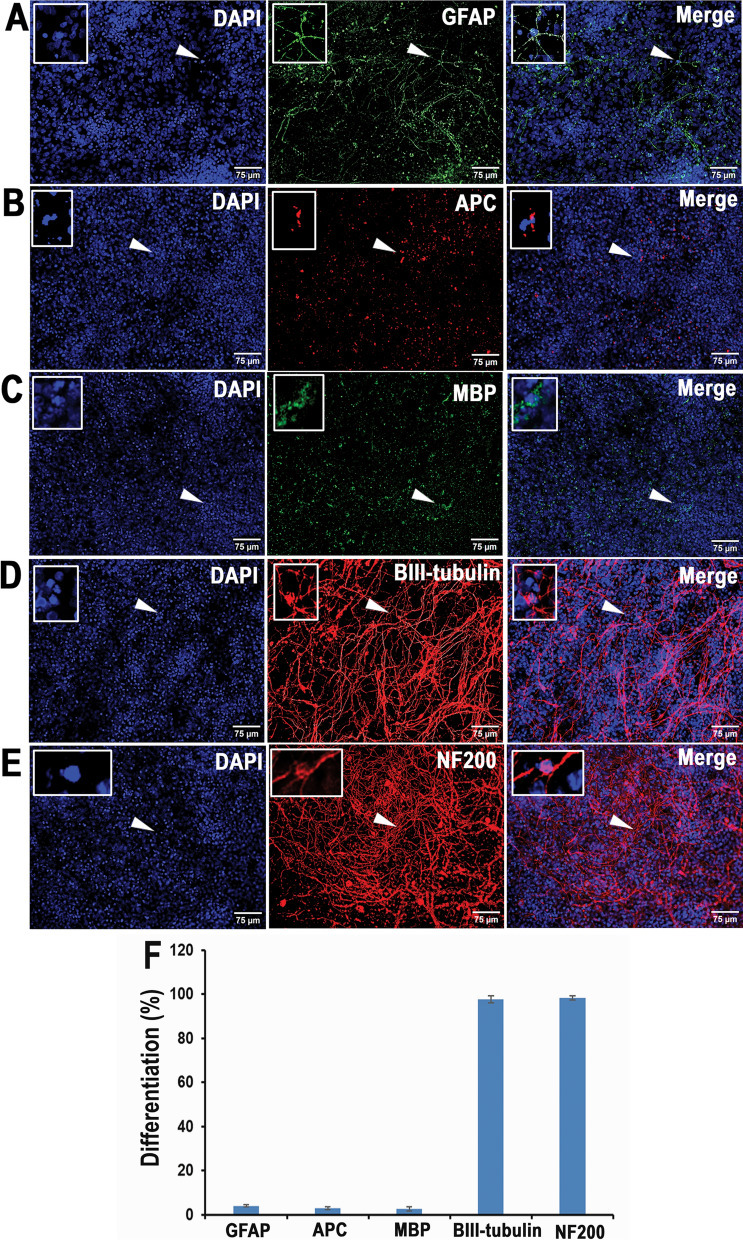
Differentiation pattern of human iPSC-derived sNPCs at 21 days in vitro. Representative images of DAPI-labeled cells with neural markers: **A** GFAP, **B** APC, **C** MBP, **D** βIII-tubulin, and **E** NF200. Scale bar: 75 μm. The arrowheads indicate the highlighted cell (Boxed) in each image. **F** Percentage of differentiated cells

Human sNPCs developed in our laboratory with defined protocols express markers that are regionally specific to the human spinal cord. This cell population consists of a mixture of immature caudal ventral spinal neurons and restricted progenitors based on HOX gene expression, ventral domain markers, motor neuron-related transcripts, and general pathways affiliated with stemness [13]. We confirmed that after 21 days these human iPSC-derived sNPCs expressed markers of neuronal lineage. The majority of the sNPCs (Fig. 1A–E and Fig. 1F) were positive for neuronal markers βIII-tubulin (96.45 ± 1.19%) and NF200 (99.2 ± 0.73%), while few cells expressed the glial markers GFAP (4.0 ± 0.57%), APC (3.3 ± 0.53%), and MBP (2.18 ± 2.18%).

### Glial scar ablation using RB was achieved in chronically injured rat spinal cords at 8 and 16 weeks (w) after transplantation

Additional file 3: Fig. S1 demonstrates the glial scar ablation method. Combinatorial treatment with RB and human iPSC-derived sNPCs (Additional file 4: Fig. S2A–E) showed no effect on the volume of the lesion cavity in chronically injured rat spinal cords at 8 w and 16 w after transplantation. Transplanted cell density was significantly (*p* < 0.05) decreased in the 1-cm area around the lesion when the glial scar was ablated (Additional file 4: Fig. S2F) demonstrating the negative effect of RB on sNPC cell survival. Hence, the findings led to the decision not to include RB induced glial scar ablation in the remaining experiments. Additional file 5: Fig. S3 depicts the influence of glial scar ablation on differentiation of the sNPCs. Further details are included in Additional file 2: Supplementary Results.

### Combinatorial treatment with human iPSC-derived sNPC transplantation and TANES has no effect on lesion cavity size

After SCI, the rat spinal cords developed a lesion cavity as expected. Sixteen weeks’ post-transplantation, the size of this cavity was compared between all three groups, and no significant difference was noted. Cells derived from the transplanted sNPCs did not show evidence of migration into the lesion cavity in either the sNPC-only or sNPC + TANES groups (Additional file 6: Fig. S4). Further, there was no additional spared tissue identified in any of these groups. Further details are included in Additional file 2: Supplementary Results.

### The addition of TANES did not affect sNPC survival and demonstrated an increased number of oligodendrocytes

**Fig. 2:**
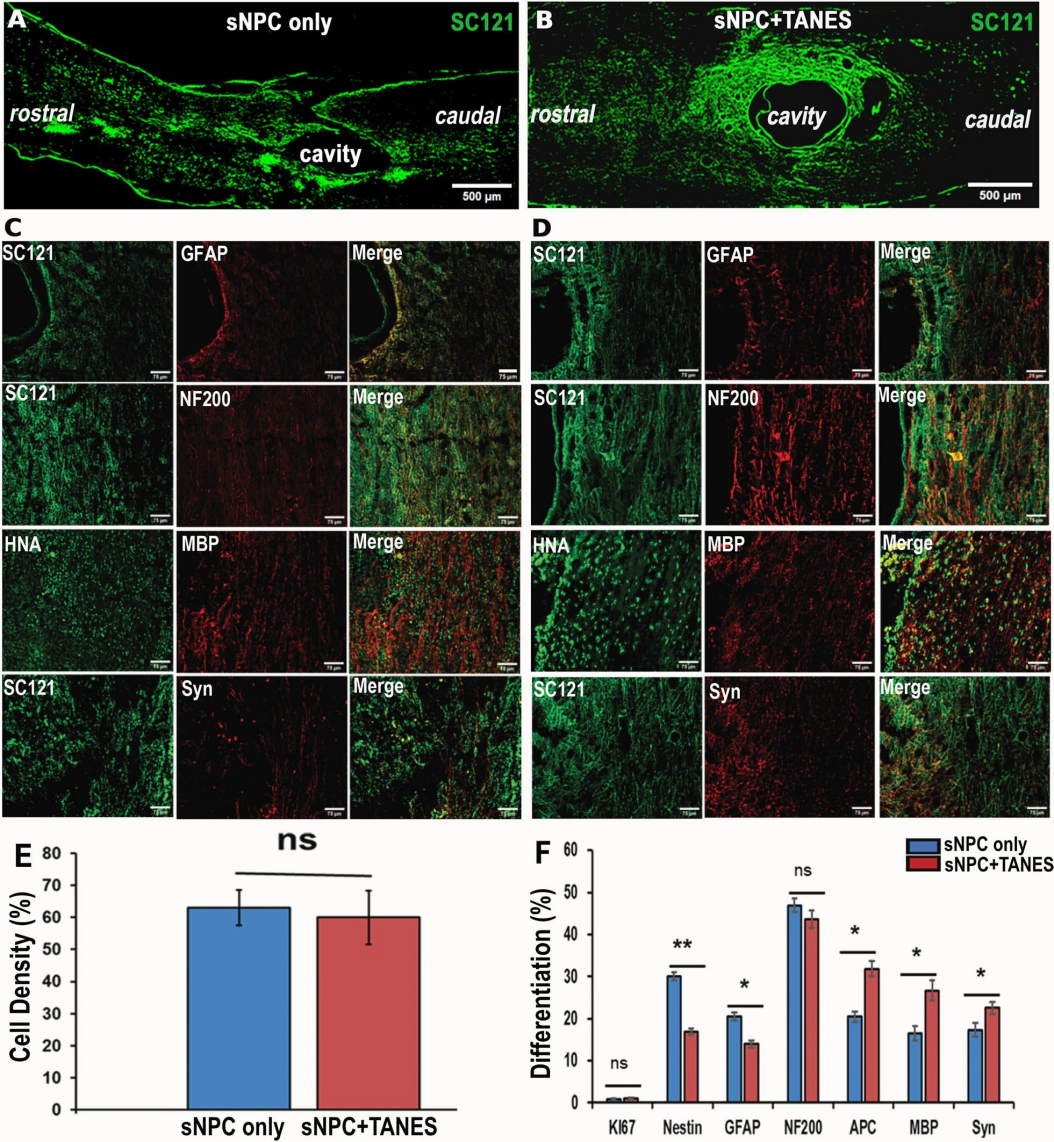
Differentiation pattern of Human iPSC-derived sNPCs with or without TANES in chronically injured spinal cord of rats. Representative images depicting the SC121^+^ cells in the rostro-caudal direction to the lesion cavity of the spinal cord **A** sNPC only and **B** sNPC + TANES. Representative images of HNA or SC121-positive cells in green double labeled with GFAP, NF200, MBP, and synaptophysin (Syn) are represented in red in both **C** sNPC-only and **D** sNPCs + TANES groups. Scale bar: **A**, **B** 500 μm, **C**, **D** 75 μm. **E** Estimation of surviving cells utilizing cell density, **F** Quantification of the percentage of co-localization of human cells with specific markers (Ki67, Nestin, GFAP, NF200, APC, MBP, and Syn) in sNPC-only and sNPC + TANES groups. Data represent mean ± standard error of the mean; *p < 0.05, **p < 0.01; ns, non-significant

To determine the fate of human iPSC-derived sNPCs post-transplantation into chronically injured rat spinal cords, we examined SC121 or HNA-positive cells co-labeled either with Ki67 (proliferation), Nestin (neural progenitors), GFAP (astrocytes), APC (mature oligodendrocytes), NF200 (neurons), MBP (myelination), or synaptophysin (synapses), as depicted in Fig. 2A, B. Also, GAT1 (GABA transporter) and VGlut1 (glutamate transporter) were analyzed and are depicted in Additional file 7: Fig. S5.

To investigate the effects of TANES on transplanted sNPC survival in chronic SCI, we compared cell density of the transplanted sNPCs at 16 weeks after transplantation in the sNPC-only and sNPC + TANES groups. We observed no statistical difference in cell survival in sNPC + TANES (60 ± 10.3%) when compared with the sNPC-only (63 ± 9.22%) group (Fig. 2C).

Further quantitative analysis of cell differentiation is presented in Fig. 2D. Sixteen weeks post-transplantation the percentage of transplanted cells that co-expressed Ki67 was very low and similar in both sNPC-only (0.9 ± 0.9%) and sNPC + TANES (1.09 ± 0.21%) groups with no statistically significant difference. These results indicated that few transplanted cells were still proliferating at the experimental end point in both treatment groups, and we also observed that the Ki67-positive cells were distributed uniformly across the engrafted area. Nestin is an indicator of cells that are not fully differentiated [30]. Transplanted cells in rats receiving TANES expressed a highly significantly (*p* < 0.01) lower percentage (17 ± 0.84%) of Nestin as compared to the sNPC-only group (30.02 ± 1.01%). This suggests that TANES encourages cell differentiation of sNPCs. Expression of the astrocytic marker GFAP was significantly (*p* < 0.05) reduced in the TANES group (13.96 ± 0.83%), as compared with the sNPC-only group (20.50 ± 1.01%), indicating that sNPCs were not differentiating into more astrocytes.

We then determined the percentage of transplanted cells demonstrating either a neuronal or oligodendrocyte fate in both the sNPC-only and sNPC + TANES groups. Many of the transplanted cells with (46.89 ± 2.08%) or without (43.68 ± 1.61%) TANES treatment expressed the neuronal marker NF200, suggesting that a neuronal fate was the most common and that this was irrespective of whether or not TANES was administered, and not different between the treatment groups. We further noticed transplanted sNPCs expressed both GAT1 as compared with VGlut1 with or without TANES. However, percentage of cells that expressed GAT1 under the influence of TANES (73.75 ± 2.08%) was significantly higher than sNPC-only group (61.02 ± 2.36%) (Additional file 7: Fig. S5). The percentage of transplanted cells that expressed oligodendrocyte markers APC and MBP in the sNPC-only and sNPC ± TANES groups was also examined. The transplanted cells that co-expressed APC were 20.61 ± 1.23% and 31.87 ± 1.83% in the sNPC-only and sNPC + TANES groups, respectively, whereas 16.45 ± 1.74% and 26.65 ± 2.37% of transplanted cells expressed MBP in the sNPC-only versus sNPC + TANES groups. This suggests that TANES not only resulted in significantly (*p* < 0.05) more oligodendrocyte differentiation, but also that these oligodendrocytes were producing myelin.

**Fig. 3:**
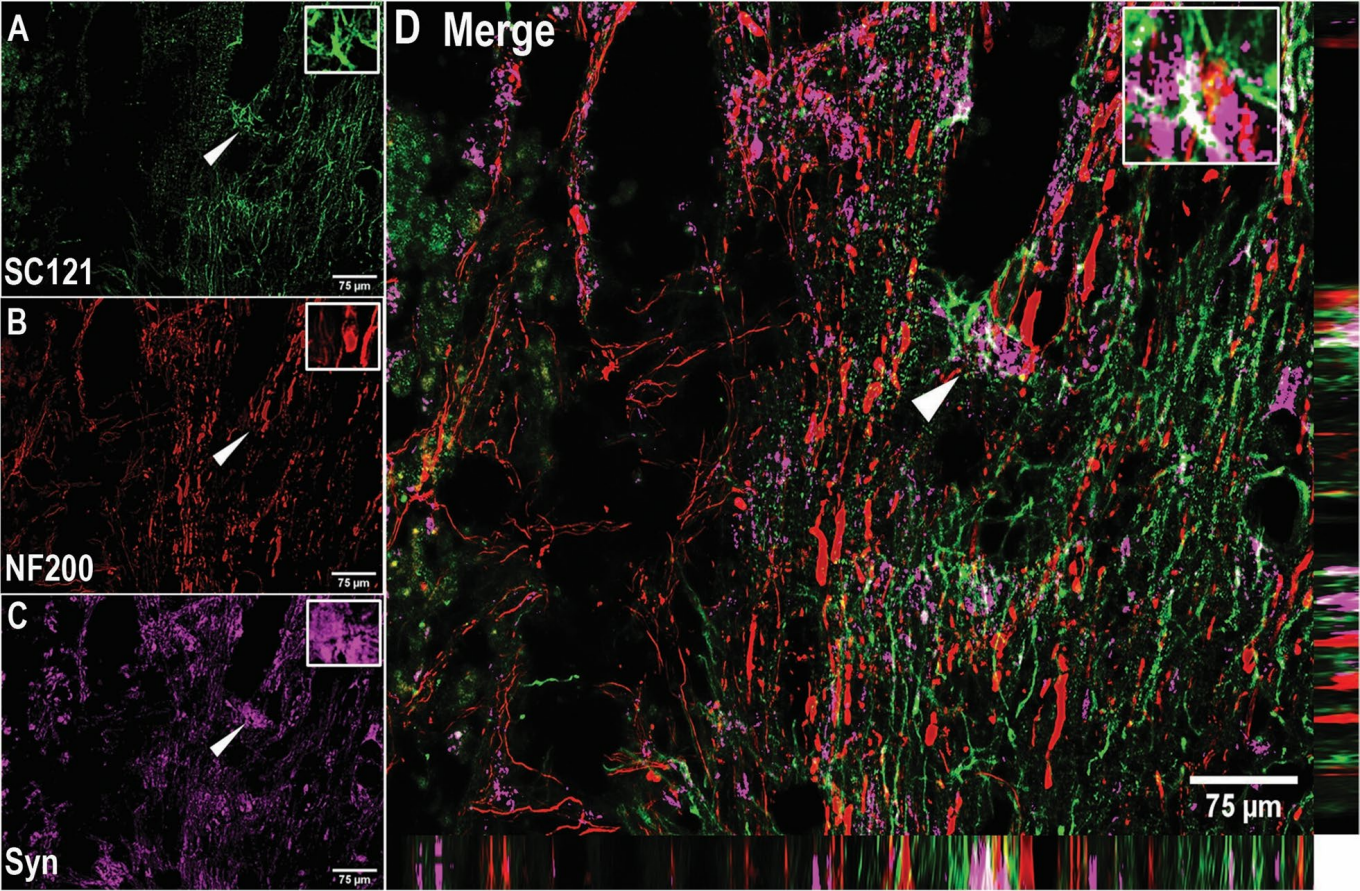
Expression of synaptophysin (Syn) around transplanted sNPCs and host neurons. Representative image of the proximity of transplanted cells, host neurons and synaptophysin. **A** SC121, **B** NF200, **C** Syn, and **D** orthogonal view of the z stack image of SC121 (green), NF200 (red) and Synaptophysin (magenta) along with the orthogonal projection. Arrowheads indicate positively labeled cells. Scale bar: 75 μm. This finding suggests synapse formation between the transplanted cells and host cells

Further, the percentage of transplanted cells that expressed synaptophysin when treated with TANES was significantly (*p* < 0.05) greater at 22.6 ± 1.41% as compared to the sNPC-only group at 17.3 ± 1.61%. This suggests an increase in multiple presynaptic connections by the application of TANES. The orthogonal representative image in Fig. 3 depicts that the synaptophysin was located at or adjacent to transplanted cells. It was noted that the expression of this presynaptic marker was often found between transplanted cells (SC121^+^) and host neurons (NF200^+^) that suggest potential synaptic connections with host neurons.

### The addition of TANES resulted in increased migration and axonal/dendritic projections of transplanted cells both rostral and caudal to the lesion site

**Fig. 4:**
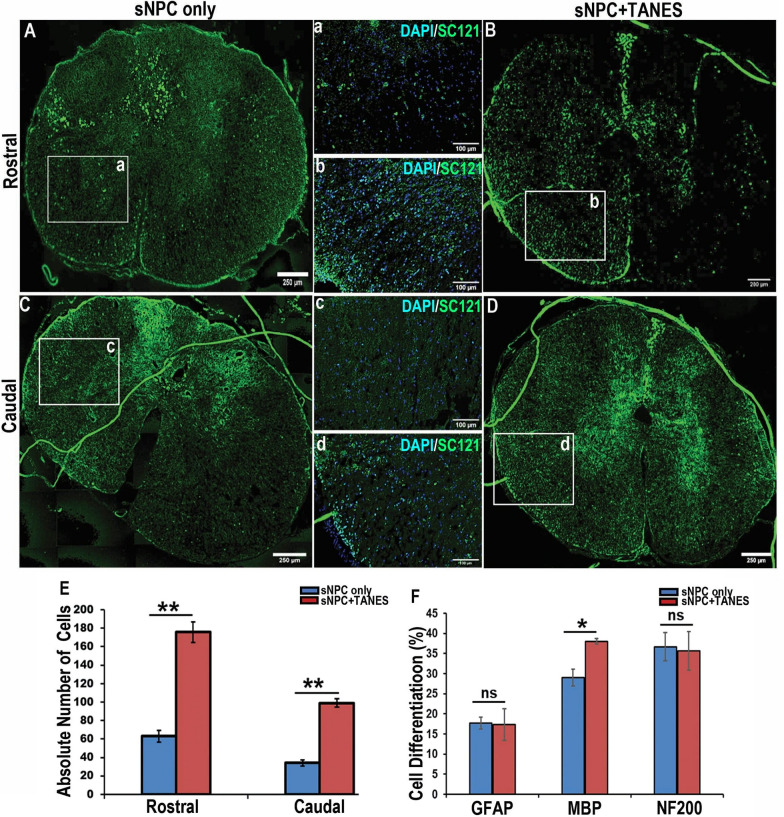
Effect of TANES on survival and migration of transplanted sNPCs both rostral and caudal to the lesion site. Spinal cords sections demonstrating HNA (green) and DAPI (blue) 16 weeks post-transplantation rostral to the lesion site **A**,** a** sNPC only, **B**, **b **sNPC + TANES; caudal to the lesion cavity **C**, **c** sNPCs only, **D**,** d** sNPCs + TANES. Scale bar: **A**–**D** 250 μm; **a**–**d** 100 μm. **E** Quantification of migrated cells at 0.6 cm rostrally and caudally to the lesion cavity, **F** Percentage of cells with specific markers GFAP, MBP, and NF200 in sNPC-only or sNPC + TANES groups at 0.6 cm rostral and caudal to the lesion cavity. Data represent mean ± standard error of the mean; *p < 0.05; **p < 0.01; ns, non-significant

Sixteen weeks post-transplantation the majority of the transplanted sNPC cell bodies were located at or adjacent to the injection sites. Axial slices of spinal cord at 0.6 cm rostral and caudal to the lesion site were also examined to determine cell migration and axonal/dendritic extension. We observed SC121-positive transplanted cells in both rostral and caudal directions in both treatment groups. However, since SC121 is a cytoplasmic marker, it was unclear whether this represented cell migration versus axonal/dendritic extension. Thus, we subsequently used the nuclear marker HNA, which was observed at both rostral and caudal locations. We noted that not all of the SC121-positive cells were also positive for HNA, suggesting that there was also axonal/dendritic extension present. Overall, more reactivity to nuclear marker HNA antibody in these rostral and caudal locations was identified in those rats that were stimulated with TANES, compared to those that were not. Counterstaining with DAPI suggested that some of these were representative of migrated cell bodies (Fig. 4A–D). The absolute number of transplanted cells (Fig. 4E) was significantly (*p* < 0.01) higher in both rostral (175.67 ± 11.07) and caudal (99.0 ± 4.75) locations in the sNPC + TANES group as compared with rostral (63.0 ± 6.38) and caudal (34 ± 2.36) directions in the sNPC-only group. Also, a higher number of HNA-positive cells were found rostrally compared to caudally in both the treatment groups. Further, we found that a significantly (*p* < 0.05) higher percentage (38.67 ± 0.67%) of sNPCs co-localized with MBP (Fig. 4F; Additional file 8: Fig. S6) in both rostral and caudal directions after TANES treatment when compared with the sNPC-only (29 ± 2.08%) group. There was no significant difference in the expression of the neuronal marker NF200 between the sNPC-only (35.67 ± 3.53%) and sNPC + TANES (35.67 ± 4.85%) groups in both the locations. A smaller percentage of the migrated cells were astrocytic, and again no significant difference was noted between sNPC only (17.67 ± 1.45%) and sNPC + TANES (17.33 ± 3.53%). Also, we investigated the effect of TANES on proliferating endogenous NSC (Additional file 9: Fig. S7). Only a small percentage of endogenous NSCs were proliferative. However, rats that were electrically stimulated expressed a higher percentage (4.2 ± 0.38%) of proliferating cells (Nestin^+^/Ki67^+^) as compared to sNPC only (2.8 ± 0.18%) and injury only (3.1 ± 0.35) toward the caudal to the injury.

To analyze the axonal/dendritic projections further away from injury site in both rostral and caudal directions, we performed exploratory tissue clearing using representative (*n* = 2) spinal cords from both treatment groups. This enabled visualization of whole spinal cord in 3D. These were subsequently immunostained with SC121 to identify the transplanted cells (Additional file 10: Fig. S8A, B). We observed that in addition to cell migration, in these randomly selected samples, the spinal cord that had been exposed to TANES demonstrated axonal/dendritic projections rostral (3.1 cm) and caudal (1.6 cm) to the injury site (Additional file 10: Fig. S8C), whereas without TANES (sNPC-only group) we noticed no detectable SC121-positive axonal/dendritic projections both rostral and caudal to the injury site in this tissue cleared sample. While no definitive conclusions can be drawn in terms of comparison, given the *n* = 1 for each group, this demonstrates that the cells have the ability to extend axonal/dendritic projections at least this distance.

### Expression of serotonin-positive fibers (5HT) was enhanced in the lumbar (L1–L3) region of the spinal cord after TANES treatment

**Fig. 5:**
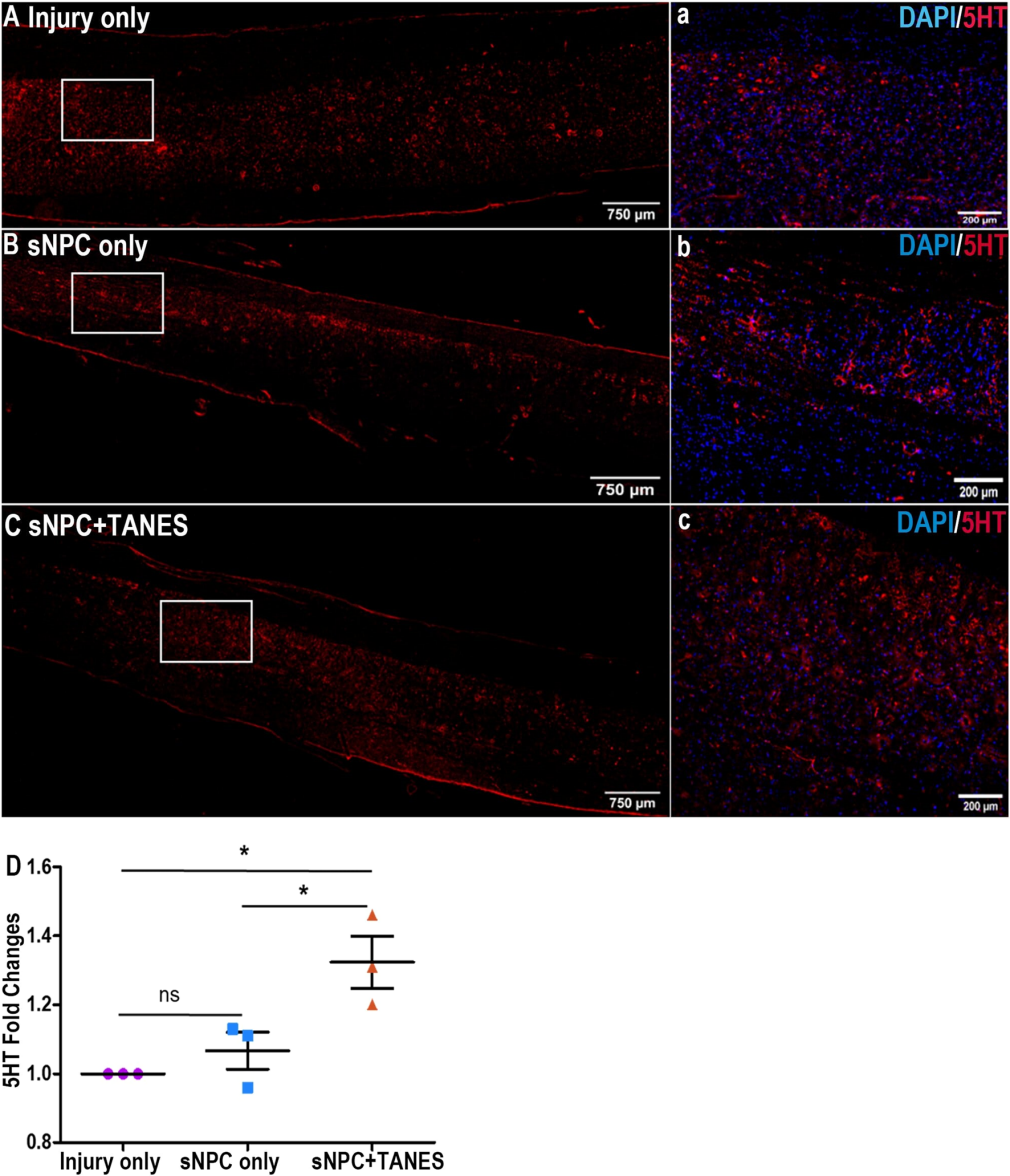
Effect of TANES on the expression of serotonin-positive fibers (5HT) in the lumbar (L1–L3) region. Spinal cords were examined for the expression of serotonin-positive fiber (5HT, red) post treatment with either **A** injury only, **B** sNPC only, or **C** sNPC + TANES. **a**–**c** Higher-magnification images of the boxed areas. Scale bars: **A**–**C** 750 μm; **a**–**c** 200 μm. **D** Quantitative analysis revealed that expression of 5HT was significantly higher when rats received sNPCs and TANES. Data represents mean ± standard error. *p < 0.05; ns, non-significant

The serotonergic pathway plays an important role in mediating descending influences on locomotion [31]. The L1–L3 levels of the rat spinal cord were examined for 5HT expression because 5HT regulates the rhythm and coordination of movements through the central pattern generator (CPG) and the L1–L2 segment corresponds to areas known to contain elements of the CPG for locomotion in rodents [32]. An increase in the expression of 5HT by 1.10 times was noted in this region in the sNPC group compared to the injury-only group, but this was not significant. However, a greater increase in the levels of the expression of 5HT by 1.27 and 1.15 times was noted in the sNPC + TANES group compared with the injury-only and sNPC-only groups, respectively, and this was significant (*p* < 0.05) (Fig. 5).

### TANES in combination with human iPSC-derived sNPCs trended toward improved functional recovery

**Fig. 6:**
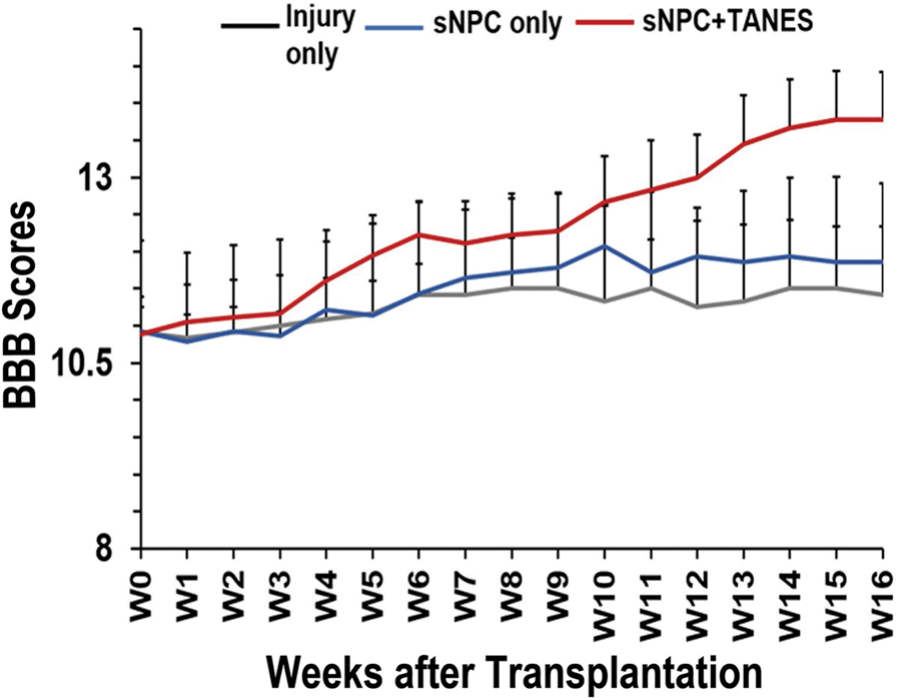
Functional recovery analysis with BBB open field locomotor scores after combinatorial treatment with sNPC transplantation and TANES. There was a slight trend in the BBB scores from week 1 to week 16 when rats transplanted with sNPCs alone were compared with rats with injury alone. A much greater trend was observed when rats were stimulated with TANES, as compared with injury only

Locomotor recovery of the hind limbs was assessed using the BBB scores (Fig. 6; Additional files 11, 12, 13: Video S1–S3). The rats in the sNPC + TANES group received TANES from week 1 through week 16 after transplantation. As expected, the injury-only group (11.41 ± 0.64) demonstrated very little improvement at week 16. The sNPC-only group (11.86 ± 0.48) showed steady, but minimal improvement as compared to the injury-only group that was not statistically significant. The sNPC + TANES group (13.78 ± 1.15) demonstrated much greater improvement than either of the other two groups, however this did not reach statistical significance. Also, we did not observe any statistical significance in the BBB sub scores among all the treatment groups.

Von Frey testing was also performed at pre-injury, pre-transplantation, 4, 8, and 16 weeks after cell transplantation to investigate the potential development of chronic pain as has been reported in other cell transplantation paradigms [33]. There was no evidence of development of chronic pain in any of the groups at any time point, regardless of treatment group (observational only).

## Discussion

It is generally accepted in the SCI community that there will be no “one approach fits all” for the treatment of SCI. Instead, there will likely be several patient and injury specific therapeutic options, some of which will be combinatorial in nature. Glial scar ablation [34], cell transplantation [35], and neuromodulation [21] are all promising approaches, and here we have attempted to explore these in combination.

The goal of our first study was to utilize a novel method of glial scar ablation to determine the effect on regionally specific transplanted spinal neural progenitor cells (sNPCs) in order to determine whether to proceed with this intervention in the next phase of this study. There is a plethora of literature to support the negative effects of glial scarring, which impedes axonal regeneration and functional recovery. Spinal cord injury (SCI) results in scar tissue formation at the lesion site and creates an inhospitable environment for axonal regeneration [36–42]. This scar consists of both glial (mainly astrocytic) and fibrotic components [42] that have been suggested to have inhibitory effects. However, the rationale for eliminating the glial scar remains controversial [43, 44]. We utilized a novel technique for glial scar ablation using the phototoxic chemical RB, which we have shown previously to be safe and effective with minimal collateral effects on functioning spared tissue. We also described an inflammatory reaction that resulted from this intervention [17, 18, 20]. We found that transplanted pre-OPCs survived and filled the lesion cavity in chronically injured rats after glial scar ablation. However, in the current study we found that glial scar ablation with RB had a detrimental effect on the survival of sNPCs (GSA + sNPC) when compared to no glial scar ablation (sNPC only) at 8 and 16 weeks after transplantation into the chronically injured rat spinal cord. The lower percentage of cell survival when treated with RB could be attributed to increased sensitivity of the transplanted sNPCs in comparison to the pre-OPCs to toxic mediators released by dead astrocytes after photoablation [45, 46]. A further explanation is that the sNPCs were more sensitive to the inflammatory environment mediated by macrophage infiltration [17].

In this first study, we also demonstrated that transplanted human iPSC-derived sNPCs can survive and differentiate 16 weeks after transplantation in this chronic model. The majority of our transplanted cells differentiated into mature neurons in all of the treatment groups. Interestingly, although in vitro almost all of the sNPCs expressed markers of neuronal lineage, the transplanted sNPC population were demonstrated to be multi-potent and retained the ability to differentiate into astrocytes, oligodendrocytes, and neurons when transplanted into chronically injured rats. Similar observations have been made by various authors, including ourselves, with different cells types and treatment paradigms [13, 18, 47–49] including using different rat strains that requires injections of cyclosporine to prevent rejection of human neural cell grafts which could drive the transplanted cells toward glial fate. We further observed that the expression of NF200 was significantly higher without glial scar ablation as compared with glial scar ablation at both 8 and 16 weeks after transplantation. This is consistent with our hypothesis that neurons are more sensitive and are dying at a proportionately higher rate than glial cells. This observation was reported previously in a study demonstrating that the ablation of scar-forming astrocytes could exacerbate neuronal cell death and demyelination following the injury as a result of an influx of blood-derived macrophages and fibrotic cells [50], which is consistent with our previous finding of an increase in macrophages post-RB scar ablation. Unlike our previous study with pre-OPCs [18], glial scar ablation with RB was detrimental to transplanted cell survival and integration into the injured chronic rat spinal cord. Therefore, we excluded glial scar ablation in the second study of combinatorial treatment with cell transplantation and electrical stimulation with TANES.

In the second study, we combined sNPC transplantation with TANES to determine whether TANES, when used in combination, affected the transplanted cells, and specifically whether they improved their integration and differentiation of the transplanted cells. There are several papers that have reported the beneficial effects of electrical stimulation [51–53]. Earlier work from our team has demonstrated that TANES alone can improve neurological outcomes in rats, and may be associated with spinal plasticity, tissue repair, and/or axonal regeneration. It has also been suggested that TANES can activate the central pattern generator (CPG) in the lumbar spinal cord to promote locomotor recovery in the contused rat spinal cord [21, 54]. Our rationale for including TANES in this protocol was to potentially optimize the connections of new neurons derived from the sNPCs. Cellular transplantation therapies alone do not always result in improvement of motor function in rats with chronic SCI [53, 54]. We therefore hypothesized that human iPSC-derived sNPCs in combination with TANES may improve cellular integration, differentiation, along with functional recovery when compared with sNPCs alone. In this study, chronically injured rats were allocated to one of three treatment groups including injury only, sNPC only, and sNPC + TANES. We examined the survival and fate of transplanted sNPCs and whether this was affected by TANES, as well as effects of TANES on the lumbar spinal cord.

Our first observation was that the volume of the lesion cavity remained similar in all of the treatment groups. Transplanted sNPCs surprisingly did not fill the lesion cavity as we had previously reported in a subacute model of SCI [12]. Instead, they were found in the spared tissue around the lesion cavity [11]. This indicates that transplanted sNPCs in a subacute model are either more attracted to the lesion site or unable to migrate in large numbers into the preserved tissue yet appear unable to survive in the cavity in a chronic model, and instead prefer the spinal cord parenchyma. As observed earlier in this study, we found that the transplanted sNPC population was multi-potent and retained the ability to differentiate into astrocytes, oligodendrocytes, and neurons when transplanted into chronically injured rats, a finding similar to our prior subacute study. The majority of our transplanted cells are differentiated into mature neurons with or without TANES. However, a higher percentage of our transplanted cells differentiated into oligodendrocytes when the rats were electrically stimulated with TANES as compared to the sNPC-only group and these cells synthesized and secreted myelin basic protein (MBP) with TANES treatment. This is a significant finding and supportive of the concept that myelin is formed preferentially on electrically active axons [55, 56]. Disruption of oligodendrocytes or the myelin sheath after SCI has severe consequences on neuronal function [57, 58]. This disruption of oligodendrocytes triggers rapid migration and proliferation of adjacent NG2^+^ cells to restore their density through a balance of active growth and self-repulsion available to replace lost oligodendrocytes and participate in tissue repair [59]. This endogenous tissue repair could be augmented by cell replacement therapy and restore local neuronal connectivity and promote the remyelination of denuded axons. Other studies have shown that endogenous, neuronal activity-dependent myelin remodeling in the adult CNS is emerging as a mechanism of CNS plasticity [60]. Our findings support the hypothesis that electrical stimulation promotes oligodendrocyte development and myelination in injured spinal cord [61–63]. This important finding could help to explain why electrical stimulation in humans can sometimes lead to permanent changes in motor function, even when the stimulation is no longer being applied [64, 65].

The formation of synapses between human iPSC-derived neurons and host rat spinal cord neurons is crucial for functional recovery given the hypothesis of relay system formation. Interestingly, we observed expression of presynaptic markers between transplanted cells (SC121^+^) and host neurons (NF200^+^) and that electrical stimulation with TANES significantly increased the amount of synaptophysin, suggesting potential synapse formation was enhanced. This is supportive of other studies that report the combination of electro‐acupuncture and the transplantation of human iPSC‐derived NPCs that subsequently differentiated into neurons and formed synaptic connections with host neurons to promote the recovery of motor function after SCI [66].

Alternatively, Li and Li [67] also noted that oligodendroglia regenerative responses could be mediated by axon-OPC synapses and electrical stimulation could increase the number of synapses. Similar observations with electro-acupuncture stimulation [66] suggest that mechanisms could include an improved local microenvironment, increased endogenous levels of NT‐3 [54], activating TrkC/AKT signaling resulting in the enhanced survival and differentiation of neurons, and promoting synapse‐like junction formation by transplanted NSCs, as well as their integration in spinal neuronal circuits.

Electrical stimulation on transplanted cells is a complicated process that is regulated by various external and internal factors. Studies have shown that electrical stimulation directly influence PI3K/Akt, mitogen-activated protein kinase (MAPK)/extracellular signal-regulated kinase (ERK) and/or Rho-kinase signaling pathways regulating NSC migration and differentiation [68–70]. Also, electrical stimulation can provide artificial stimulation that transmits electrical charge to the cells that are electrically active [56].

Interestingly we also observed that the electrical stimulation with TANES significantly increased the expression of GABA transporter. Previous studies [71, 72] have shown that human spinal GABA neurons could mitigate hindlimb spasticity and improve locomotion.

Other significant findings are that 30% of the transplanted cells remained in an immature state as evident from the expression of the immature cell marker Nestin after 16 weeks, consistent with what has been previously described [30, 73]. Treatment with TANES significantly reduced the number of immature transplanted cells. Transplanted cells also retained the ability to form astrocytes in both groups, albeit slightly but significantly less when electrically stimulated with TANES. Finally, a small percentage of transplanted cells expressed Ki67, which indicated that few of the cells were still in a proliferative state 16 weeks’ post-transplantation in all groups; this is also consistent with previous studies [11, 74].

We observed migration of the transplanted human iPSC-derived sNPCs in chronically injured rats in both of the treatment groups. The neuronal cell bodies were generally localized around the lesion site [11]. A higher number of transplanted cell bodies were found rostrally to the lesion as compared to caudally in both treatment groups. Application of TANES not only promoted a significantly higher number of sNPCs found remotely from the site of injection but also influenced long-distance axonal/dendritic projections especially rostrally (3.1 cm) to the lesion site as compared to treatment without TANES. As Salazar et al. [11] has suggested, this preferential rostral extension might be due to the rostral cord still receiving connections from the brain potentially contributing to a more trophic environment for transplanted cells. Other studies have found evidence for enhanced cell migration toward the source of current, known as electrotaxis [75], but we did not find this in our current study.

To further explore the effects of TANES, we examined the expression of serotonergic neurons at the lumbar (L1–L3) region. Endogenous plasticity by axon growth/ sprouting of serotonergic axons has been observed in different SCI models in mice and rats by various researchers [76–78] but with severe injuries there has been a failure to restore locomotor function without the use of additional therapeutic interventions. It has been demonstrated that the descending serotonergic tracts directly modulate locomotion function and SCI affects activation of the CPG which can result in subsequent depletion of 5HT [79, 80]. Perrin et al. [81] suggest that functional consequences of SCI at the thoracic level can be improved by a substitutive transplantation of 5HT neurons or regeneration under the trophic influence of grafted stem cells that are capable of activating both TrkB and C receptors in a rodent model. We found that rats transplanted with human iPSC-derived sNPCs, when treated with sNPCs in combination with TANES resulted in an increased expression of serotonergic neurons.

While there were no significant differences between treatment groups, there were trends that suggested a likely underpowered study for functional improvement, and this will be further investigated with larger studies. Trends toward improvement in BBB scores to consistent coordinated stepping in the sNPC + TANES group is notable.

In summary, the focus of this study was firstly to determine whether the glial scar ablation that we were exploring was a positive addition to our combinatorial therapy, and these results were negative as increased cell death resulted. The second focus was to determine whether the addition of electrical stimulation to sNPC transplantation had significant effects, and these results were more positive.

## Conclusions

Together, the data suggest that combinatorial treatment with human iPSC-derived sNPCs and electrical stimulation with TANES may provide a novel platform to promote cellular integration in chronically injured rats. The microenvironment that was created by electrical stimulation influenced the fate of human iPSC-derived sNPCs transplanted into the injured spinal cord. These sNPCs were shown to be multi-potent and were more likely to differentiate into cells of the oligodendrocyte lineage and increase both myelination and synapse formation when rats were electrically stimulated with TANES. We suggest that activity-dependent oligodendrocyte and myelin remodeling from the transplanted cells along with the neuroplasticity induced by TANES increases connectivity and indicates a potential mechanism for functional recovery.

## Supplementary Information

**Additional file 1. Supplementary Table 1.** Randomized allocation of rats to perform various experiments in the study.

**Additional file 2.** Supplementary methods, results, and animal handling and monitoring documentation.

**Additional file 3. Supplementary Figure. 1.** Expression of glial fibrillary acidic protein (GFAP) post-glial scar ablation (GSA) in chronically injured spinal cord of rats 8 and 16 weeks (w) after transplantation. GFAP expression (red) around the lesion cavity in rats receiving either (A, B) sNPC only (8 and 16w), or (C, D) GSA+sNPC (8w and 16w). Scale bar: 200μm. The circle denotes the lesion boundary. (E) Quantitative analysis of GFAP expression demonstrated that scar ablation with rose Bengal significantly reduces the amount of GFAP. Data represent mean± standard error of the mean; *p<0.05.

**Additional file 4. Supplementary Figure 2.** Cavitation analysis post-glial scar ablation in chronically injured rat spinal cord 8 and 16 weeks (w) after transplantation. Representative images of the transplanted cells were identified with an antibody against SC121 represented in green (A–B) sNPC only (8w and 16w), or (C–D) GSA+sNPC (8w and 16w). Scale bar: 50μm. (E) Volume of the lesion cavity demonstrated that the GSA+sNPC group did not result in a decrease in the volume of the cavity compared with the sNPCs only groups both at 8w and 16w after transplantation. The circle denotes the lesion boundary. (F) Quantification of percentage of cell density. Data represent mean± standard error of the mean; ***p<0.001; ns, non-significant.

**Additional file 5. Supplementary Figure 3.** Differentiation of Human iPSC derived-sNPCs with and without glial scar ablation (GSA) in chronically injured rat spinal cord 8 and 16 weeks (w) after transplantation. Representative images of the transplanted cells were either identified with an antibody against SC121 or HNA, represented in green. These cells were double labeled with (A) Ki67, (B) Nestin, (C) GFAP, (D) APC, or (E) NF200 represented in red in both sNPC only and GSA+sNPC groups at 8w and 16w after transplantation. Scale bar: 50μm. (a-e) Percentage of co-localization of SC121+/ HNA+ cells with specific markers in sNPC only or GSA+sNPC groups at 8w and 16w after transplantation. Data represent mean± standard error of the mean; *p<0.05; **p<0.01; ns, non-significant.

**Additional file 6. Supplementary Figure 4.** Histology and cavitation analysis post-TANES in chronically injured rat spinal cord 16 weeks after sNPC transplantation. Representative images of hematoxylin and eosin/Luxol fast blue staining of parasagittal sections of spinal cord from rats subjected to (A) Injury only, (B) sNPC only and (C) sNPC+TANES. Scale bar: 300μm. (D) Area of the lesion cavity demonstrated that there was no significant difference in any of the groups 16week after transplantation. Data represents mean± standard error of the mean, ns, non-significant.

**Additional file 7. Supplementary Figure 5.** Effect of TANES on differentiation of Human iPSC-derived sNPCs into GAT1 and VGlut1 in chronically injured rat spinal cord after transplantation. Representative images of the transplanted cells identified with an antibody against SC121 (green). These cells were double labeled with (A-B) GAT1 or (C-D) VGlut1 represented in red in both sNPC only and GSA+sNPC groups. Scale bar: 75μm. (E) Percentage of co-localization of SC121+ cells with specific markers in sNPC only or GSA+sNPC groups after transplantation. Data represent mean± standard error of the mean; *p<0.05; ns, non-significant.

**Additional file 8. Supplementary Figure 6.** Effect of TANES on the differentiation pattern of Human iPSC derived- sNPCs that migrated rostral and caudal to the lesion site. Representative images of the transplanted cells identified with an antibody against SC121 (green) double labelled with GFAP, MBP and NF200 are represented in red. (A-C) sNPC only rostral to the lesion cavity, (a-c) sNPC only caudal to the lesion cavity, (D-E) sNPC+TANES rostral to the lesion cavity and (d-e) sNPC+TANES caudal to the lesion site. Scale bar: 150μm.

**Additional file 9. Supplementary Figure 7.** Effect of TANES on the proliferation of endogenous NPCs residing in the central canal rostral and caudal to the lesion site. Spinal cord sections demonstrating merged images of Nestin (green), Ki67 (red) and DAPI (blue) rostral to the lesion cavity (A) Injury only, (B) sNPC only and (C) sNPC+TANES. Caudal to lesion cavity (D) Injury only, (E) sNPC only and (F) sNPC+TANES. Scale bar: 75μm. Higher magnification images are depicted in the boxes of the respective image with (i) Nestin, (ii) Ki67 and (iii) Merged. (G) Percentage of Nestin positive cells co-localized with Ki67 in Injury only, sNPC only or sNPC+TANES groups at 0.6cm rostrl and caudal to the lesion cavity. Data represent mean± standard error of the mean; *p<0.05.

**Additional file 10. Supplementary Figure 8.** Visualization of axonal/dendritic projections in the spinal cord using tissue clearing. Expression of SC121 represented in green in the whole spinal cord (A) rostral; (a-d) Higher magnification images at 1.6cm-3.1cm rostral to the injury site, (B) Caudal; (e-f) Higher magnification images at 1.6cm-2.1cm caudal to the injury site, 16 weeks after sNPC transplantation and TANES. Scale bar: (A, B) 100μm, (a-f) 50μm. (C) Quantification of relative integrated density per area of SC121 positive projections over the distance.

**Additional file 11. Supplementary Video 1.** Representative video of open field locomotor testing for the Injury only group taken at 16 weeks post-transplantation. This animal received a BBB score of 11.

**Additional file 12. Supplementary Video 2.** Representative video of open field locomotor testing for the sNPC only group taken at 16 weeks post-transplantation. This animal received a BBB score of 11.

**Additional file 13. Supplementary Video 3.** Representative video of open field locomotor testing for the sNPC+TANES group taken at 16 weeks post-transplantation. This animal received a BBB score of 13.

## Data Availability

The data that support the findings of this study are available from the corresponding author upon reasonable request. A long-term data sharing, and preservation plan will be used to store and make publicly accessible the data beyond the life of the project. The data will be deposited into the Data Repository for the University of Minnesota (DRUM), http://hdl.handle.net/11299/166578.

## Abbreviations

ANOVA: Analysis of variance
BBB: Basso–Beattie–Bresnahan
DAPI: 4′,6-Diamidino-2-phenyl indole
DPBS: Dulbecco’s phosphate-buffered Saline
GFAP: Glial fibrillary acid protein
GSA: Glial scar ablation
H&E: Hematoxylin and eosin
HNA: Human nuclear antigen
ICC: Immunocytochemistry
IHC: Immunohistochemistry
iPSC: Induced pluripotent stem cells
LFB: Luxol fast blue
MBP: Myelin basic protein
NF200: Neurofilament
PBS: Phosphate-buffered saline
SCI: Spinal cord injury
SEM: Standard error of the mean
sNPCs: Spinal neuronal progenitor cells
TANES: Tail nerve electrical stimulation

## References

[1] https://www.nscisc.uab.edu/Public/Facts%20and%20Figures%202020.pdf

[2] Zhang S, Huang F, Gates M, White J, Holmberg EG. Tail nerve electrical stimulation induces body weight-supported stepping in rats with spinal cord injury. J Neurosci Methods. 2010;187(2):183–9. 20079372 10.1016/j.jneumeth.2010.01.008

[3] Darrow D, Balser D, Netoff T, Krassioukov A, Phillips A, Parr A, Samadani U. Epidural spinal cord stimulation facilitates immediate restoration of dormant motor and autonomic supraspinal pathways after chronic neurologically complete spinal Cord Injury. J Neurotrauma. 2019;36(15):2325–36. 30667299 10.1089/neu.2018.6006 PMC6648195

[4] Kim TH, Kim S, Lee S, Lee JP, Snyder EY, Kook IP. Human neurospheres derived from the fetal central nervous system are regionally and temporally specified but are not committed. Exp Neurol. 2006;199(1):222–35. 16714017 10.1016/j.expneurol.2006.03.015

[5] Cheng H, Huang Y, Yue H, Fan Y. Electrical stimulation promotes stem cell neural differentiation in tissue engineering. Stem Cells Int. 2021;20:6697574. 10.1155/2021/6697574 PMC8081629 33968150

[6] Walsh P, Truong V, Hill C, Stoflet ND, Baden J, Low WC, et al. Defined culture conditions accelerate small-molecule-assisted neural induction for the production of neural progenitors from human-induced pluripotent stem cells. Cell Transpl. 2017;26(12):1890–902. 10.1177/0963689717737074 PMC5802631 29390875

[7] Parr AM, Walsh PJ, Truong V, Dutton JR. cGMP-compliant expansion of human iPSC cultures as adherent monolayers. Methods Mol Biol. 2016;1357:221–9. 25863788 10.1007/7651_2015_243

[8] Bonner JF, Connors TM, Silverman WF, Kowalski DP, Lemay MA, Fischer I. Grafted neural progenitors integrate and restore synaptic connectivity across the injured spinal cord. J Neurosci. 2011;31(12):4675–86. 21430166 10.1523/JNEUROSCI.4130-10.2011 PMC3148661

[9] Lu P, Ceto S, Wang Y, Graham L, Wu D, Kumamaru H, et al. Prolonged human neural stem cell maturation supports recovery in injured rodent CNS. J Clin Invest. 2017;127(9):3287–99. 28825600 10.1172/JCI92955 PMC5669577

[10] Rosenzweig ES, Lu P, Kumamaru H, Salegio EA, Kadoya K, et al. Restorative effects of human neural stem cell grafts on the primate spinal cord. Nat Med. 2018;24(4):484–90. 29480894 10.1038/nm.4502 PMC5922761

[11] Salazar DL, Uchida N, Hamers FPT, Cummings BJ, Anderson AJ, et al. Human neural stem cells differentiate and promote locomotor recovery in an early chronic spinal cord injury NOD-scid mouse model. PLoS ONE. 2010;5(8): e12272. 20806064 10.1371/journal.pone.0012272 PMC2923623

[12] Dell’Anno MT, Xingxing W, Onorati M, Li M, Talpo F, Sekine F, et al. Human neuroepithelial stem cell regional specificity enables spinal cord repair through a relay circuit. Nature Comm. 2018;9(1):3419. 10.1038/s41467-018-05844-8 PMC6109094 30143638

[13] Lavoie N, Truong V, Malone D, Pengo T, Patil N, Dutton JR, et al. Human induced pluripotent stem cells integrate, create synapses and extend long axons after spinal cord injury. J Cell Mol Med. 2022;26:1932–42. 35257489 10.1111/jcmm.17217 PMC8980929

[14] Fitch MT, Silver J. CNS injury, glial scars, and inflammation: Inhibitory extracellular matrices and regeneration failure. Exp Neurol. 2008;209(2):294–301. 17617407 10.1016/j.expneurol.2007.05.014 PMC2268907

[15] McKillop WM, Dragam M, Schedl A, Brown A. Conditional Sox9 ablation reduces chondroitin sulfate proteoglycan levels and improves motor function following spinal cord injury. Glia. 2013;61(2):164–77. 23027386 10.1002/glia.22424 PMC4853194

[16] Silver J. The glial scar is more than just astrocytes. Exp Neurol. 2016;286:147–9. 27328838 10.1016/j.expneurol.2016.06.018

[17] Patil N, Truong V, Holmberg MH, Lavoie NS, McCoy MR, Dutton JR, et al. Safety and efficacy of rose Bengal derivatives for glial scar ablation in chronic spinal cord injury. J Neurotrauma. 2019;35:1745–54. 10.1089/neu.2017.5398 PMC6033306 29373946

[18] Patil N, Walsh P, Carrabre K, Holmberg EG, Lavoie N, Dutton JR, et al. Regionally specific human pre-oligodendrocyte progenitor cells produce both oligodendrocytes and neurons after transplantation in a chronically injured spinal cord rat model after glial scar ablation. J Neurotrauma. 2021;38:777–88. 33107383 10.1089/neu.2020.7009

[19] Charan J, Kantharia ND. How to calculate sample size in animal studies? Pharmacol Pharmacother. 2013;4(4):303–6. 10.4103/0976-500X.119726 PMC3826013 24250214

[20] Zhang S, Kluge B, Huang F, Nordstrom T, Dolooen S, Gross M, et al. Photochemical scar ablation in chronically contused spinal cord of rat. J Neurotrauma. 2007;24:411–20. 17376003 10.1089/neu.2006.0065

[21] Zhang S, Huang F, Gates M, Holmberg EG. Tail nerve electrical stimulation combined with scar ablation and neural transplantation promotes locomotor recovery in rats with chronically contused spinal cord. Brain Res. 2012;1456:22–35. 22516110 10.1016/j.brainres.2012.03.054

[22] Basso DM, Beattie MS, Bresnahan JC. A sensitive and reliable locomotor rating scale for open field testing in rats. J Neurotrauma. 1995;12(1):1–21. 7783230 10.1089/neu.1995.12.1

[23] Lankhorst AJ, Duis SE, ter Laak MP, Joosten EA, Hamers FP, Gispen WH. Functional recovery after central infusion of alpha-melanocyte-stimulating hormone in rats with spinal cord contusion injury. J Neurotrauma. 1999;16(4):323–31. 10225218 10.1089/neu.1999.16.323

[24] Kostich W, Hamman BD, Li YW, Naidu S, Dandapani K, Fenget J, et al. Inhibition of AAK1 kinase as a novel therapeutic approach to treat neuropathic pain. J Pharma Exp Ther. 2016;383(2):371–86. 10.1124/jpet.116.235333 PMC4998676 27411717

[25] Xu L, Ryu J, Hiel H, Menon A, Aggarwal A, Rha E, et al. Transplantation of human oligodendrocyte progenitor cells in an animal model of diffuse traumatic axonal injury: survival and differentiation. Stem Cell Res Ther. 2015;6(1):93. 25971252 10.1186/s13287-015-0087-0 PMC4453242

[26] Chung K, Wallace J, Kim SY, Kalyanasundaram S, Andalman AS, Davidson TJ, et al. Structural and molecular interrogation of intact biological systems. Nature. 2013;497(7449):332–7. 23575631 10.1038/nature12107 PMC4092167

[27] Abercrombie M. Estimation of nuclear population from microtome sections. Anat Rec. 1946;94(2):239–47. 21015608 10.1002/ar.1090940210

[28] Zwolak P, Dudek AZ, Bodempudi VD, Nguyen J, Hebbel RP, Gallus NJ, et al. Local irradiation in combination with bevacizumab enhances radiation control of bone destruction and cancer-induced pain in a model of bone metastases. Int J Cancer. 2008;122:681–8. 17943718 10.1002/ijc.23157

[29] McDonald JW, Liu XZ, Qu Y, Liu S, Mickey SK, Turetsky D, et al. Transplanted embryonic stem cells survive, differentiate and promote recovery in injured rat spinal cord. Nat Med. 1999;5:1410–2. 10581084 10.1038/70986

[30] Cao QL, Howard RM, Dennison JB, Whittemore SR. Differentiation of engrafted neuronal-restricted precursor cells is inhibited in the traumatically injured spinal cord. Exp Neurol. 2002;177:349–59. 12429182 10.1006/exnr.2002.7981

[31] Bowker RM, Westlund KN, Sullivan MC, Wilber JF, Coulter JD. Descending serotonergic, peptidergic and cholinergic pathways from the raphe nuclei: a multiple transmitter complex. Brain Res. 1983;288:33–48. 6198030 10.1016/0006-8993(83)90079-3

[32] Nishimaru H, Takizawa H, Kudo N. 5-Hydroxytryptamine-induced locomotor rhythm in the neonatal mouse spinal cord in vitro. Neurosci Lett. 2000;280:187–90. 10675792 10.1016/s0304-3940(00)00805-3

[33] Hofstetter CP, Holmström NA, Lilja JA, Schweinhardt P, Hao J, Spenger C. Allodynia limits the usefulness of intraspinal neural stem cell grafts; directed differentiation improves outcome. Nat Neurosci. 2005;8:346–53. 15711542 10.1038/nn1405

[34] Tran AP, Warren PM, Silver J. Biology of regeneration failure and success after spinal cord injury. Physiol Rev. 2018;98(2):881–817. 29513146 10.1152/physrev.00017.2017 PMC5966716

[35] Angelo AD, Ionescu RB, Kazak G. The role of neural stem cells in regulating glial scar formation and repair. Cell Tissue Res. 2022;387(3):399–414. 34820704 10.1007/s00441-021-03554-0 PMC8975756

[36] Bahr M, Przyrembel C, Bastmeyer M. Astrocytes from adult rat optic nerves are nonpermissive for regenerating retinal ganglion cell axons. Exp Neurol. 1995;131:211–20. 7895822 10.1016/0014-4886(95)90043-8

[37] Davies SJ, Ghahramani P, Jackson PR, Noble TW, Hardy PG, Hippisley-Cox J, et al. Association of panic disorder and panic attacks with hypertension. Am J Med. 1999;107:310–6. 10527031 10.1016/s0002-9343(99)00237-5

[38] Hara M, Takayasu MW, Watanabe K, Noda A, Takagi T, Suzuki Y, et al. Protein kinase inhibition by fasudil hydrochloride promotes neurological recovery after spinal cord injury in rats. J Neurosurg. 2000;93:94–101. 10879764 10.3171/spi.2000.93.1.0094

[39] Raposo C, Schwartz M. Glial scar and immune cell involvement in tissue remodeling and repair following acute CNS injuries. Glia. 2014;62(11):1895–904. 24756949 10.1002/glia.22676

[40] Li Z, Yu S, Hu X, Li Y, You X, Tian D, et al. Fibrotic scar after spinal cord Injury: Crosstalk with other cells, cellular origin, function, and mechanism. Front Cel Neurosci. 2021;15:1–12. 10.3389/fncel.2021.720938 PMC8441597 34539350

[41] Quraishe S, Forbes LH, Andrews MR. The extracellular environment of the CNS: influence on plasticity, sprouting, and axonal regeneration after spinal cord injury. Neural Plast. 2018;2018:2952386. 29849554 10.1155/2018/2952386 PMC5932463

[42] Silver J, Miller JH. Regeneration beyond the glial scar. Nat Rev Neurosci. 2004;5:146–56. 14735117 10.1038/nrn1326

[43] Faulkner JR, Hermann JE, Woo MJ, Tansey KE, Doan NB, Sofroniew MV, et al. Reactive astrocytes protect tissue and preserve function after spinal cord injury. J Neurosci. 2004;3:2143–55. 10.1523/JNEUROSCI.3547-03.2004 PMC6730429 14999065

[44] Anderson MA, Burda JE, Ren Y, Ao Y, O’Shea TM, Kawaguchi R, et al. Astrocyte scar formation aids central nervous system axon regeneration. Nature. 2016;532:195–200. 27027288 10.1038/nature17623 PMC5243141

[45] Lee YC, Park CK, Kim MS, Kim JH. In vitro study for staining and toxicity of rose Bengal on cultured bovine corneal endothelial cells. Cornea. 1996;15(4):376–85. 8776564 10.1097/00003226-199607000-00008

[46] Land W. Postischemic reperfusion injury to allografts: A case for ‘innate immunity’? Eur Surg Res. 2002;34:160–9. 11867918 10.1159/000048904

[47] Kondo T, Raff M. Oligodendrocyte precursor cells reprogrammed to become multipotential CNS stem cells. Science. 2000;289:1754–7. 10976069 10.1126/science.289.5485.1754

[48] Kim SD, Jung JS, Lee SJ, Lim BY, Kim HA, Yoo JE, et al. Rapid generation of OPC-like cells from human pluripotent stem cells for treating spinal cord injury. Exp Mol Med. 2017;49: e361. 28751784 10.1038/emm.2017.106 PMC5565952

[49] Xu T, Li X, Guo Y, Uhlin E, Holmberg L, Mitra S, et al. Multiple therapeutic effects of human neural stem cells derived from induced pluripotent stem cells in a rat model of post-traumatic syringomyelia. EBioMedicine. 2022;77: 103882. 35182996 10.1016/j.ebiom.2022.103882 PMC8857569

[50] Adams KL, Gallo V. The diversity and disparity of the glial scar. Nat Neurosci. 2018;21(1):9–15. 29269757 10.1038/s41593-017-0033-9 PMC5937232

[51] Liu LQ, Moody J, Traynor M, Dyson S, Gall A. A systematic review of electrical stimulation for pressure ulcer prevention and treatment in people with spinal cord injuries. J Spinal Cord Med. 2014;37(6):703–18. 24969965 10.1179/2045772314Y.0000000226 PMC4231958

[52] Lai BQ, Zeng X, Han WT, Che MT, Ding Y, Li G, et al. Stem cell-derived neuronal relay strategies and functional electrical stimulation for treatment of spinal cord injury. Biomaterials. 2021;279: 121211. 34710795 10.1016/j.biomaterials.2021.121211

[53] Karamian BA, Siegel N, Nourie B, Serruya MD, Heary RF, James SH, et al. The role of electrical stimulation for rehabilitation and regeneration after spinal cord injury. J Ortho Traumato. 2022;23(2):2–17. 10.1186/s10195-021-00623-6 PMC8738840 34989884

[54] Malloy DC, Knikou M, Côté MP. Adapting human-based transcutaneous spinal cord stimulation to develop a clinically relevant animal model. J Clin Med. 2022;11(7):2023. 35407636 10.3390/jcm11072023 PMC8999945

[55] McTigue DM, Horner PJ, Stokes BT, Gage FH. Neurotrophin-3 and brain-derived neurotrophic factor Induce oligodendrocyte proliferation and myelination of regenerating axons in the contused adult rat spinal cord. J Neurosci. 1998;18(14):5354–65. 9651218 10.1523/JNEUROSCI.18-14-05354.1998 PMC6793495

[56] Wake H, Ortiz FC, Woo DH, Lee PR, Angulo MC, Fields RD. Nonsynaptic junctions on myelinating glia promote preferential myelination of electrically active axons. Nature Comm. 2012;6:7844. 10.1038/ncomms8844 PMC4532789 26238238

[57] Nashmi R, Fehlings MG. Mechanisms of axonal dysfunction after spinal cord injury: with an emphasis on the role of voltage-gated potassium channels. Brain Res Rev. 2001;38:165–91. 11750932 10.1016/s0165-0173(01)00134-5

[58] Monje M. Myelin plasticity and nervous system function. Annu Rev Neurosci. 2018;41:61–76. 29986163 10.1146/annurev-neuro-080317-061853

[59] Hughes EG, Kang SH, Fukaya M, Bergles DE. Oligodendrocyte progenitors balance growth with self-repulsion to achieve homeostasis in the adult brain. Nat Neurosci. 2013;16(6):668–76. 23624515 10.1038/nn.3390 PMC3807738

[60] Faria DO, Gonsalvez DG, Nicholson M, Xiao J. Activity-dependent central nervous system myelination throughout life. J Neurochem. 2019;148(4):447–61. 30225984 10.1111/jnc.14592 PMC6587454

[61] Li Q, Brus-Ramer M, Martin JH, McDonald JW. Electrical stimulation of the medullary pyramid promotes proliferation and differentiation of oligodendrocyte progenitor cells in the corticospinal tract of the adult rat. Neurosci Lett. 2010;479:128–33. 20493923 10.1016/j.neulet.2010.05.043 PMC2922017

[62] Li Q, Houdayar T, Liu S, Belegu V. Induced neural activity promotes an oligodendroglia regenerative response in the injured spinal cord and improves motor function after spinal cord injury. J Neurotrauma. 2016;34(24):3351–61. 10.1089/neu.2016.4913 28474539

[63] Li G, Fan ZK, Gu GF, Jia ZQ, Zhang QQ, Dai JY, He SS. Epidural spinal cord stimulation promotes motor functional recovery by enhancing oligodendrocyte survival and differentiation and by protecting myelin after spinal cord injury in rats. Neurosci Bull. 2020;36(4):372–84. 31732865 10.1007/s12264-019-00442-0 PMC7142180

[64] Peña PI, Hoover C, Venkatesh S, Ahmadi A, Sturtevant D, Patrick N, et al. Long-term spinal cord stimulation after chronic complete spinal cord injury enables volitional movement in the absence of stimulation. Front Syst Neurosci. 2020;14:35. 32714156 10.3389/fnsys.2020.00035 PMC7340010

[65] Darrow D, Balser DY, Freeman D, Pelrine E, Krassioukov A, Phillips A, et al. Effect of epidural spinal cord stimulation after chronic spinal cord injury on volitional movement and cardiovascular function: study protocol for the phase II open label-controlled E-STAND trial. BMJ Open. 2022;12(7): e059126. 35851008 10.1136/bmjopen-2021-059126 PMC9297213

[66] Ding Y, Xu HO, Zeng H, Zeng X, Lai BQ, Li G, et al. Electro-acupuncture and its combination with adult stem cell transplantation for spinal cord injury treatment: a summary of current laboratory findings and a review of literature. CNS Neurosci Ther. 2022;28(5):635–47. 35174644 10.1111/cns.13813 PMC8981476

[67] Li DC, Li Q. Electrical stimulation of cortical neurons promotes oligodendrocyte development and remyelination in the injured spinal cord. Neural Regen Res. 2017;12(10):1613–5. 29171422 10.4103/1673-5374.217330 PMC5696838

[68] Dong ZY, Pei Z, Wang YI, Li Z, Khan A, Meng XT. Ascl1 regulates electric field-induced neuronal differentiation through PI3K/Akt pathway. Neuroscience. 2019;404:141–52. 30771509 10.1016/j.neuroscience.2019.02.004

[69] Meng X, Arocena M, Penninger J, Gage FH, Zhao M, Song B. PI3K mediated electrotaxis of embryonic and adult neural progenitor cells in the presence of growth factors. Exp Neurol. 2011;227(1):210–7. 21092738 10.1016/j.expneurol.2010.11.002 PMC3821524

[70] Arocena M, Zhao M, Collinson JM, Song B. A time-lapse and quantitative modelling analysis of neural stem cell motion in the absence of directional cues and in electric fields. J Neurosci Res. 2010;88(15):3267–74. 20890991 10.1002/jnr.22502

[71] Gong C, Zheng X, Guo F, Wang Y, Zhang S, Chen J, et al. Human spinal GABA neurons alleviate spasticity and improve locomotion in rats with spinal cord injury. Cell Rep. 2021;34: 108889. 33761348 10.1016/j.celrep.2021.108889

[72] Zheng X, Zhu B, Xu J, Liu D, Huang Y, Daiqi C, et al. Human spinal GABA neurons survive and mature in the injured nonhuman primate spinal cord. Stem Cell Rep. 2023;18:439–48. 10.1016/j.stemcr.2022.12.016 PMC9969075 36669493

[73] Ogawa Y, Sawamoto K, Miyata T, Miyao S, Watanabe M, Nakamura M, et al. Transplantation of in vitro-expanded fetal neural progenitor cells results in neurogenesis and functional recovery after spinal cord contusion injury in adult rats. J Neurosci Res. 2002;69:925–33. 12205685 10.1002/jnr.10341

[74] Webber DJ. Adult neural precursor cells and the dysmyelinated spinal cord. J Neurosci. 2007;27:6605–6. 17581947 10.1523/JNEUROSCI.2125-07.2007 PMC6672687

[75] Jahanshahi A, Schonfeld L, Janssen ML, Janssen F, Hescham S, Kocabicak E, et al. Electrical stimulation of the motor cortex enhances progenitor cell migration in the adult rat brain. Exp Brain Res. 2013;231:165–77. 24002672 10.1007/s00221-013-3680-4

[76] Bregman BS, McAtee M, Dai HN, Kuhn PL. Neurotrophic factors increase axonal growth after spinal cord injury and transplantation in the adult rat. Exp Neurol. 1997;1997(148):475–94. 10.1006/exnr.1997.6705 9417827

[77] Ramón-Cueto A, Plant GW, Avila J, Bunge MB. Long-distance axonal regeneration in the transected adult rat spinal cord is promoted by olfactory ensheathing glia transplants. J Neurosci. 1998;18:3803–15. 9570810 10.1523/JNEUROSCI.18-10-03803.1998 PMC6793168

[78] Zörner B, Bachmann LC, Filli L, Kapitza S, Gullo M, Bolliger M, et al. Chasing central nervous system plasticity: the brainstem’s contribution to locomotor recovery in rats with spinal cord injury. Brain. 2014;137:1716–32. 24736305 10.1093/brain/awu078

[79] Hochman S, Garraway SM, Machacek DW, Shay BL. 5-HTreceptors and the neuromodulatory control of spinal cord function. In: Cope TC, editor. Motor neurobiology of the spinal cord. London: CRC Press; 2001. p. 47–87.

[80] Pflieger JF, Clarac F, Vinay L. Postural modifications and neuronal excitability changes induced by short-term serotonin depletion during neonatal development in the rat. J Neurosci. 2002;22:5108–17. 12077206 10.1523/JNEUROSCI.22-12-05108.2002 PMC6757731

[81] Perrin FE, Gerber YN, Teigell M, et al. Anatomical study of serotonergic innervation and 5-HT (1A) receptor in the human spinal cord. Cell Death Dis. 2011;2: e218. 21993394 10.1038/cddis.2011.98 PMC3219094

