# Supplementary material for: Electrical stimulation affects the differentiation of transplanted regionally specific human spinal neural progenitor cells (sNPCs) after chronic spinal cord injury
Source: Stem Cell Res Ther. 2023 Dec 20;14:378. doi: 10.1186/s13287-023-03597-w (PMC10734202; doi:10.1186/s13287-023-03597-w)
Supplement: Supplementary file 1 — Additional file 1. Supplementary Table 1. Randomized allocation of rats to perform various experiments in the study. [file 13287_2023_3597_MOESM1_ESM.docx]

**Additional File 1**

**Supplementary Table 1: Randomized allocation of rats to perform various experiments in the study**

| **Objective** | **Total # of rats with expected attrition** | **Estimated # of rats for statistical significance** | **Treatment**  **groups** | **Lost due to post- operative**  **complications** | **Exclusions**  **due to inconsistent injury (> 210kdyn)** | **Functional studies** | **Tissue utilized for**  **Histology IHC assay** | **Tissue Clearing assay** |
| --- | --- | --- | --- | --- | --- | --- | --- | --- |
| Aim 1 | n=24  20% attrition | n=20  10 rats/group | sNPC only  (8w and 16w)  n=12 | n=1 | n=3 | NA | n=5 |  |
|  |  |  | GSA+sNPC  (8w and 16w)  n=12 |  |  | NA | n=5 |  |
| Aim 2 | n=36  30% attrition | n=24  8 rats/group | Injury only  n=12 | n=4 | n=5 | n=9 | n=5 | NA |
|  |  |  | sNPC only  n=12 |  |  | n=9 | n=5 | n=1 |
|  |  |  | sNPC+TANES  n=12 |  |  | n=9 | n=5 | n=1 |

n= # of rats

NA=not applicable
