# Supplementary material for: Electrical stimulation affects the differentiation of transplanted regionally specific human spinal neural progenitor cells (sNPCs) after chronic spinal cord injury
Source: Stem Cell Res Ther. 2023 Dec 20;14:378. doi: 10.1186/s13287-023-03597-w (PMC10734202; doi:10.1186/s13287-023-03597-w)
Supplement: Supplementary file 2 — Additional file 2. Supplementary methods, results, and animal handling and monitoring documentation. [file 13287_2023_3597_MOESM2_ESM.docx]

**Additional File 2**

**Supplementary Methods**

**Von Frey Test**

Rats were acclimatized for 1 hour to the experimental room and then placed into observational boxes with wire-mesh floors for 20 minutes. Paw withdrawal threshold was recorded using a series of von Frey hairs with a force of 0.6 g, 2.0 g, 4.0 g, 8.0 g, 15.0 g, 26.0 g, 60.0 g applied perpendicularly to the midplantar surface ^3^. Brisk paw withdrawal or licking immediate after pressure application was defined as a positive response, and lack of paw withdrawal within 6 seconds was defined as a negative response. Ambulation was considered an ambiguous response, and in such cases the stimulus was repeated. Rats from all the treatment groups were evaluated for paw withdrawal threshold performed at weeks 4, 8, and 16 after cell transplantation.

**Supplementary Results**

**Treatment with Rose Bengal (RB) Ablates the Glial Scar in Chronically Injured Rat Spinal Cords at 8 and 16 weeks (w) after Transplantation**

Immunohistrochemistry was performed to determine the effects of rose Bengal (RB) on scar ablation in chronically injured rat spinal cords post-treatment with RB. In this study we confirmed our previous findings that the glial scar was ablated with RB administration (Additional file 1: Supplementary Fig. 1A-D). The integrated density quantified the expression of GFAP around the injury epicenter (Supplementary Fig. 1C). The results demonstrated that GFAP/astrocytes were significantly reduced when the scar was ablated with RB in the GSA+sNPC group (6.5×10^5^) compared with the sNPC only (9.3×10^5^) group at 8w. A similar trend was observed at 16w (sNPC:10.0×10^5^; GSA+sNPC:7.1×10^5^). The results suggest that GFAP positive astrocytes were considerably decreased when treated with RB, consistent with our previous publication.

**Combinatorial Treatment with Rose Bengal (RB) and Human iPSC-Derived sNPCs do not Affect the Volume of the Lesion Cavity in Chronically Injured Rat Spinal Cords at 8 and 16 weeks (w) after Transplantation**

Twelve weeks after SCI, the glial scar was photo ablated with RB. Histological analysis was performed 16 weeks after cell transplantation. Parasagittal sections surrounding the lesion cavity were subjected to H&E/LFB staining and representative images are depicted in Supplementary Fig. 2. The glial scar appears as a layer of continuous tissue around the lesion cavity when chronically injured spinal cords were not treated with RB in the sNPC only (8w and 16w) groups (Supplementary Fig. 2A-B), and this glial scar was mostly ablated when treated with RB in the GSA+Snpc (both at 8w and 16w) group as shown in Supplementary Fig.2C-D.

Quantitative analysis of the total volume of the cavity was performed (Additional file 1: Supplementary Fig. 2E). The total cavity volume in the sNPC only group was 0.82mm^3^ at 8w and 0.87mm^3^ at 16w. The total cavity volume in the GSA+sNPCs group at 8w and 16w after transplantation was 0.86mm^3^ and 0.85mm^3^, respectively. This observation suggests that the environment created by glial scar ablation with RB did not result in sNPCs filling the lesion cavity in the GSA+sNPC only group at either 8w or 16w.

Further, to determine the effect of glial scar ablation on the survival of the transplanted cells (Supplementary Fig. 2F), the human specific antibody HNA was utilized. Significant differences were observed in the average percentage of cell survival at 8w in the sNPC only group (30.0±2.0%) compared to the GSA+sNPC (15.04+1.6%) group. RB treatment was detrimental to the transplanted cells as there was a significant difference in survival at 8w and also at 16w in the sNPC only group (28.5±1.4%) versus the GSA+sNPC (11.3±0.80%) group.

**Treatment with Rose Bengal (RB) Affects the Differentiation of Human iPSC-Derived sNPCs in Chronically Injured Rat Spinal Cords at 8 and 16 Weeks (w) after Transplantation**

To determine the fate of human iPSC-derived sNPCs after transplantation at 8w and 16w, we examined SC121/HNA positive cells co-labeled either with Ki67 (proliferation), Nestin (neural progenitors), GFAP (astrocytes), APC (oligodendrocytes), or NF200 (neurons/axons) as depicted in Supplementary Fig. 3A-E. The quantitative analysis is presented in Supplementary Fig 3a-e.

Sixteen weeks’ post-transplantation, the percentage of HNA positive cells that co-expressed with Ki67 was very low among all the groups. There was no statistically significant difference between the groups: sNPC only (8w:1.94±0.11%;16w:2.15±0.10%) and GSA+sNPC (8w:2.17±0.54%; 16w:2.6±0.65%). These results indicated that few transplanted cells were still proliferating at the experimental end point in both treatment groups, and we also observed that the Ki67 positive cells were distributed uniformly across the engrafted area. Some transplanted cells retained an immature phenotype shown by Nestin expression in both treatment groups (8w:36.8±3.54%; 16w:33.6±1.43%) in the sNPC only group and (8w:36.63±9.51%; 16w:37.07±9.27%) in the GSA+sNPC group. Quantitative analysis showed that there was no statistically significant difference between the two groups at 8w and 16w after transplantation. Similarly, there was no significant difference in the expression of GFAP (astrocytes) in both the sNPC only (8w:14.67±0.52%;16w:14.85±0.63%) and the GSA+sNPC (8w:14.90±3.72%; 16w:15.0± 3.75%) group.

We determined the percentage of transplanted cells demonstrating either neuronal or oligodendroglial fate. The majority of the transplanted cells expressed neuronal markers. We utilized neuronal markers (NF200). Eight weeks after cell transplantation there was no significant difference in the expression of NF200 between the sNPC only (39.84±1.04%) and the GSA+sNPC (32.17±8.04%) group. We then quantified the expression of NF200 at 16w after cell transplantation and the quantitative analysis revealed that the percentage of transplanted cells expressing the neuronal marker was significantly greater in the sNPC only group (45.82±0.89%) when compared with the GSA+sNPC group (31.44±7.85%).

Further, a minority of the cells expressed oligodendrocytic markers. No difference was observed in the percentage of APC at 8w in the sNPC only group (2.45±0.19%) and GSA+sNPC group (3.13±0.78%). However, transplanted cells showed a significant difference in the expression of APC between the two treatment groups in the sNPC only (2.01+0.24%) and GSA+sNPC (3.1±1.55%) groups with a higher percentage of cells expressing markers of oligodendrocyte lineage at 16w when the glial scar was ablated. This suggests when the glial scar was not ablated, a higher percentage of transplanted cells differentiated into neurons compared with cells transplanted into glial scar ablated cords. Our original proposal was that sNPCs will have superior survival and integration into the injured spinal cord when used in combination with glial scar ablation. However, the results showed glial scar ablation with rose Bengal had no beneficial effect on sNPC survival and integration into chronically injured cords, and in fact was detrimental. Therefore, we excluded the glial scar ablation in the combinatorial treatment with sNPC transplantation and TANES.

**Animal Handling and Monitoring**

The study was performed in strict accordance with the recommendations in the Guide for the Care and Use of Laboratory Animals of the National Institutes of Health (NIH). The protocol was approved by the Institutional Animal Care and Use Committee at the University of Minnesota (Protocol Number 1810-36461A). We selected rats because they have become standard laboratory animals in testing of functional recovery after spinal cord injury. Behavioral testing has been thoroughly described and validated in rats. Animals were monitored daily to determine whether they are listless and have stopped eating and drinking. Surgical sites were examined carefully for signs of infection. When bladders are expressed (twice daily until bladder recovery), the urine was examined for gross hematuria. Animals were weighed once a week at the time of the functional testing for weight loss. The following clinical signs will be monitored: failure to groom, lethargy, dyspnea, persistent self-trauma. Rats were observed daily. RAR consulted if the rat needs to be euthanized for humane reasons. Athymic nude rats were observed to see if any fur has fallen in their eyes. Monitoring was included weekends and holidays. Immediately after surgery, our rats were singly housed to prevent them from licking or biting at the fresh wounds. When we remove the sutures at 2 weeks, we are then typically re-house them in pairs.
