# Supplementary figures and images for: Electrical stimulation affects the differentiation of transplanted regionally specific human spinal neural progenitor cells (sNPCs) after chronic spinal cord injury
Source: Stem Cell Res Ther. 2023 Dec 20;14:378. doi: 10.1186/s13287-023-03597-w (PMC10734202; doi:10.1186/s13287-023-03597-w)

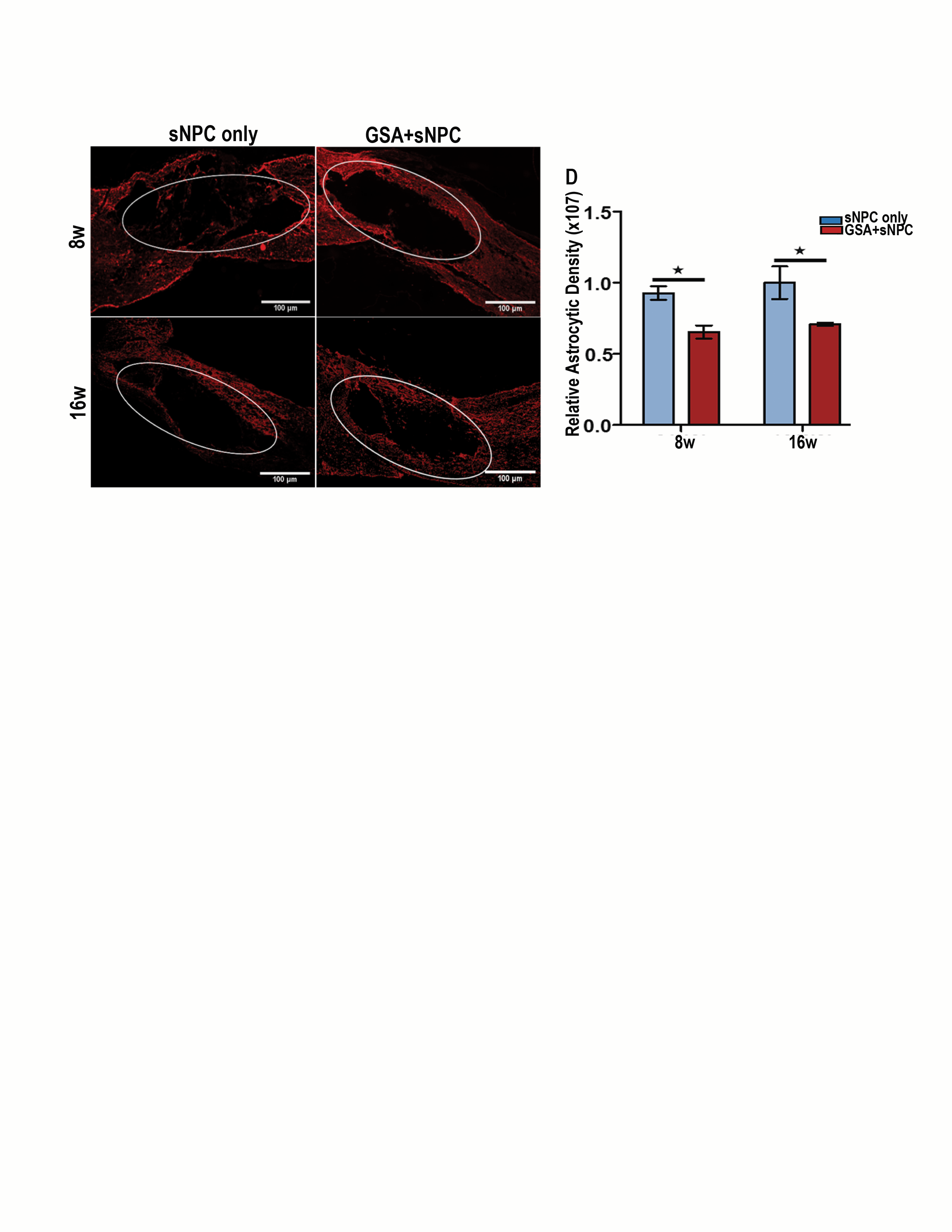

Supplement: Supplementary file 3 — Additional file 3. Supplementary Figure. 1. Expression of glial fibrillary acidic protein (GFAP) post-glial scar ablation (GSA) in chronically injured spinal cord of rats 8 and 16 weeks (w) after transplantation. GFAP expression (red) around the lesion cavity in rats receiving either (A, B) sNPC only (8 and 16w), or (C, D) GSA+sNPC (8w and 16w). Scale bar: 200μm. The circle denotes the lesion boundary. (E) Quantitative analysis of GFAP expression demonstrated that scar ablation with rose Bengal significantly reduces the amount of GFAP. Data represent mean± standard error of the mean; *p<0.05. [file 13287_2023_3597_MOESM3_ESM.tiff]

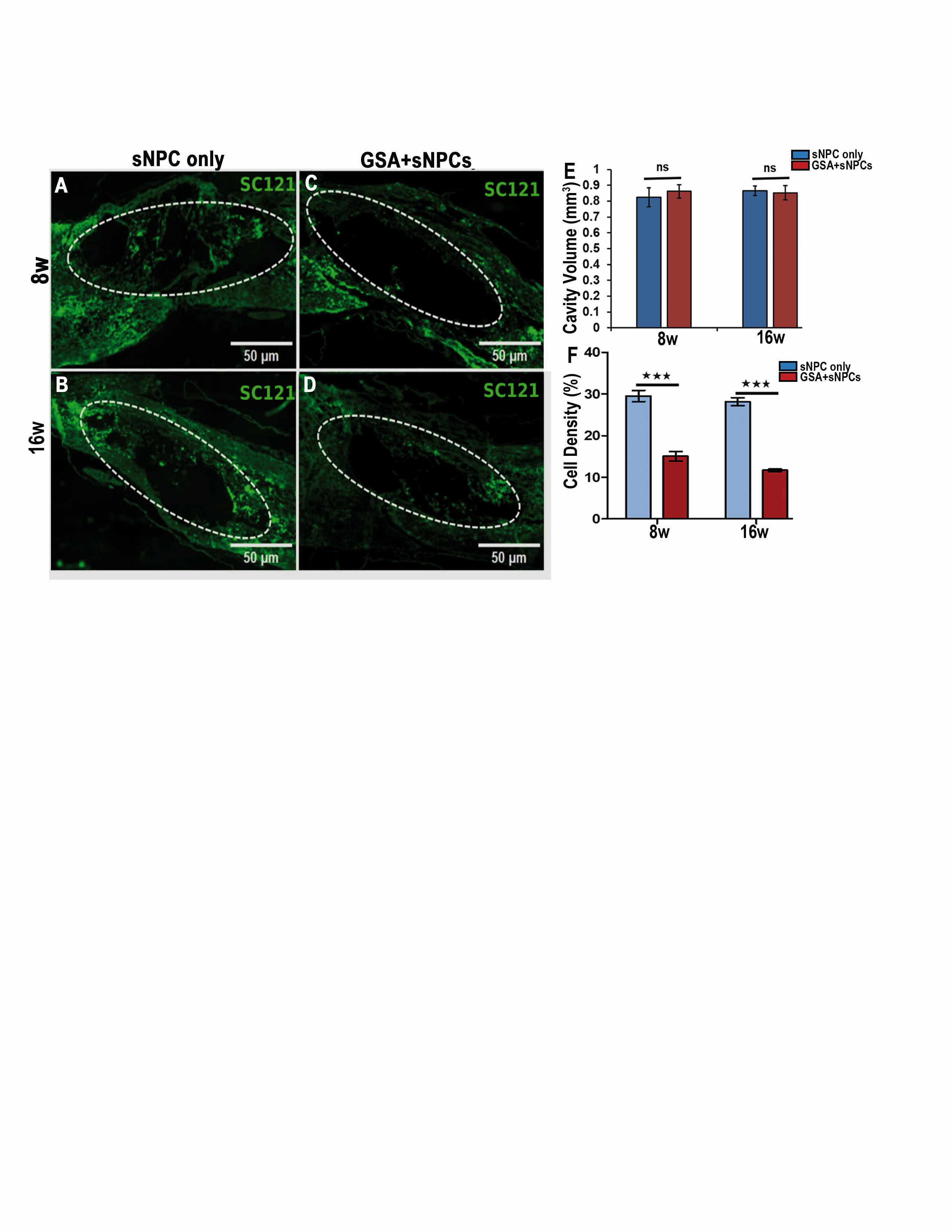

Supplement: Supplementary file 4 — Additional file 4. Supplementary Figure 2. Cavitation analysis post-glial scar ablation in chronically injured rat spinal cord 8 and 16 weeks (w) after transplantation. Representative images of the transplanted cells were identified with an antibody against SC121 represented in green (A–B) sNPC only (8w and 16w), or (C–D) GSA+sNPC (8w and 16w). Scale bar: 50μm. (E) Volume of the lesion cavity demonstrated that the GSA+sNPC group did not result in a decrease in the volume of the cavity compared with the sNPCs only groups both at 8w and 16w after transplantation. The circle denotes the lesion boundary. (F) Quantification of percentage of cell density. Data represent mean± standard error of the mean; ***p<0.001; ns, non-significant. [file 13287_2023_3597_MOESM4_ESM.tiff]

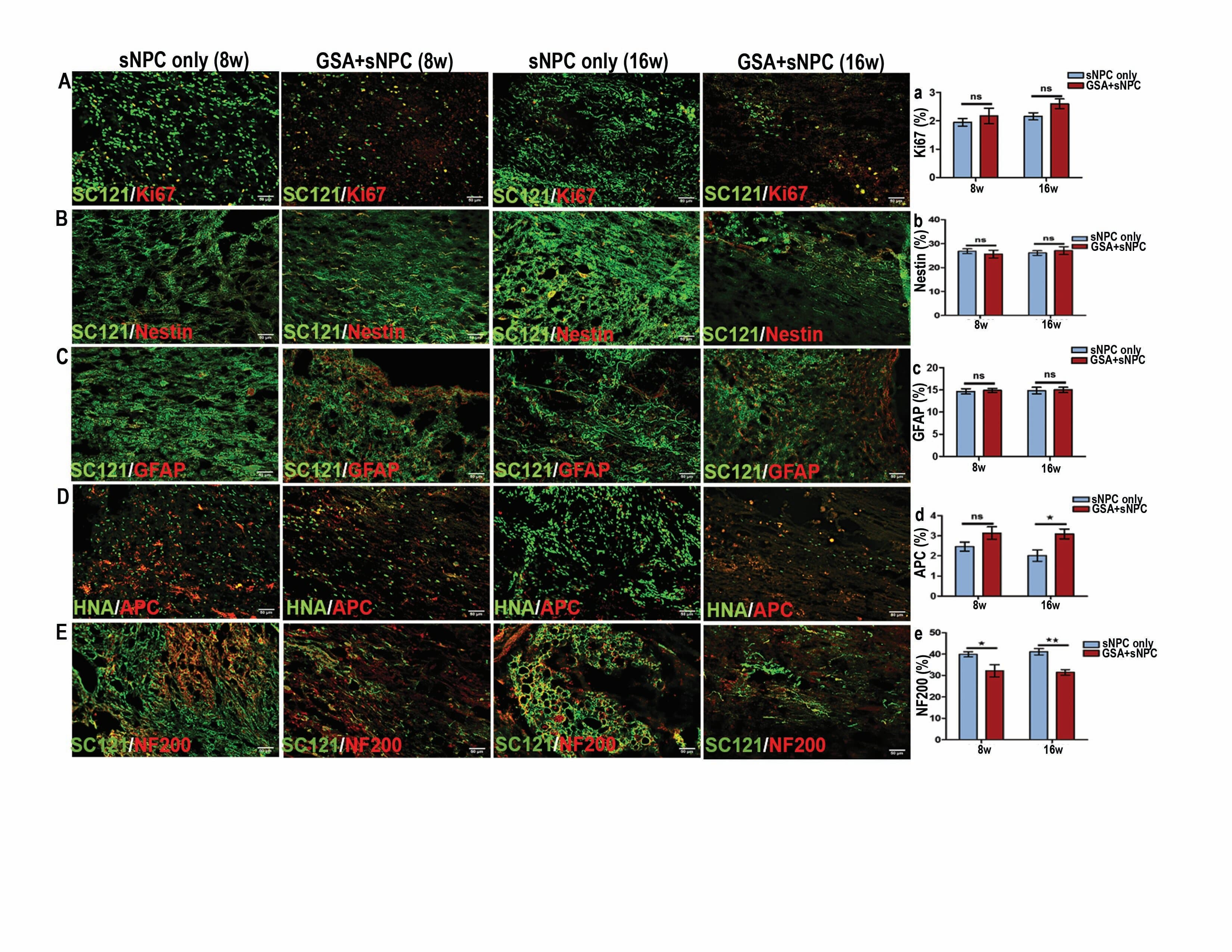

Supplement: Supplementary file 5 — Additional file 5. Supplementary Figure 3. Differentiation of Human iPSC derived-sNPCs with and without glial scar ablation (GSA) in chronically injured rat spinal cord 8 and 16 weeks (w) after transplantation. Representative images of the transplanted cells were either identified with an antibody against SC121 or HNA, represented in green. These cells were double labeled with (A) Ki67, (B) Nestin, (C) GFAP, (D) APC, or (E) NF200 represented in red in both sNPC only and GSA+sNPC groups at 8w and 16w after transplantation. Scale bar: 50μm. (a-e) Percentage of co-localization of SC121+/ HNA+ cells with specific markers in sNPC only or GSA+sNPC groups at 8w and 16w after transplantation. Data represent mean± standard error of the mean; *p<0.05; **p<0.01; ns, non-significant. [file 13287_2023_3597_MOESM5_ESM.tiff]

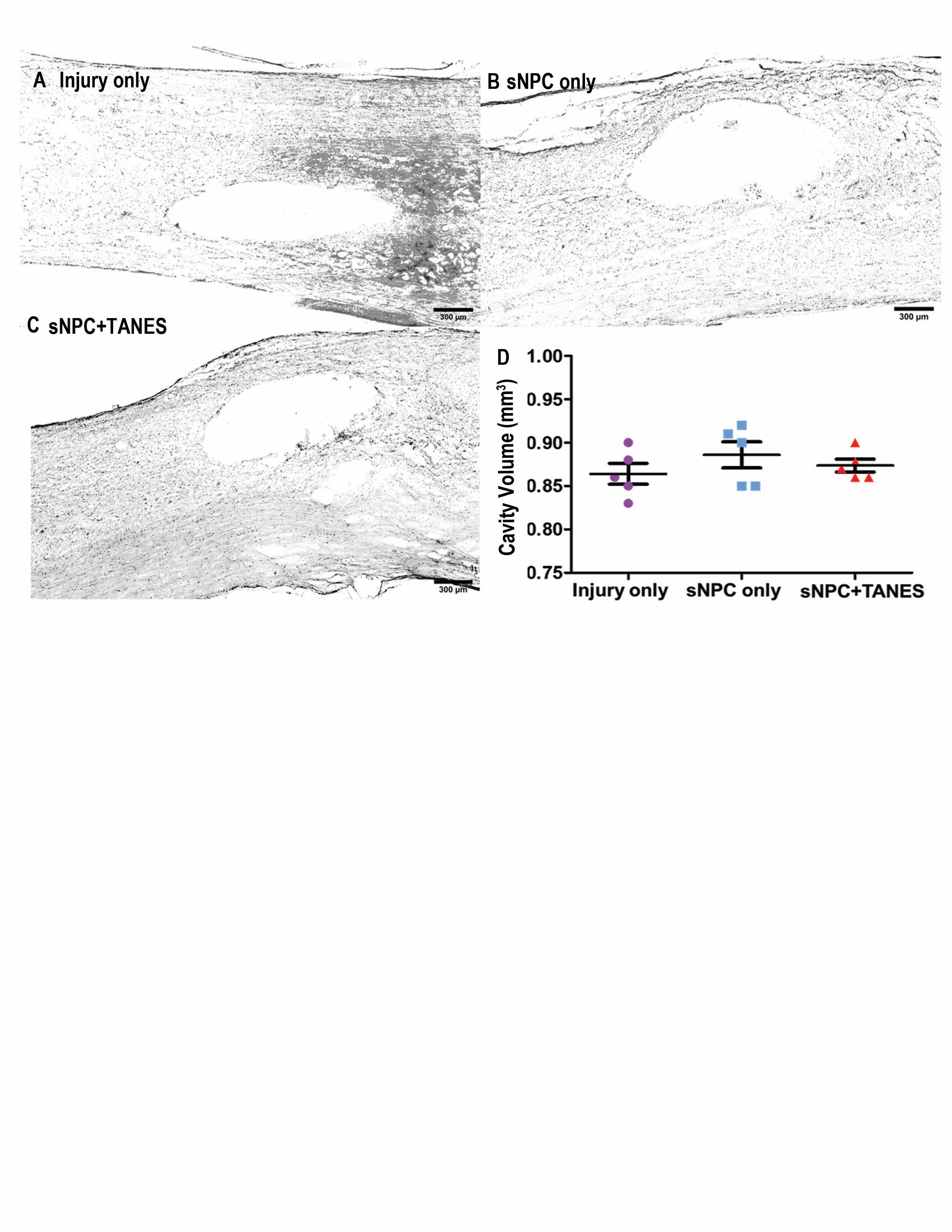

Supplement: Supplementary file 6 — Additional file 6. Supplementary Figure 4. Histology and cavitation analysis post-TANES in chronically injured rat spinal cord 16 weeks after sNPC transplantation. Representative images of hematoxylin and eosin/Luxol fast blue staining of parasagittal sections of spinal cord from rats subjected to (A) Injury only, (B) sNPC only and (C) sNPC+TANES. Scale bar: 300μm. (D) Area of the lesion cavity demonstrated that there was no significant difference in any of the groups 16week after transplantation. Data represents mean± standard error of the mean, ns, non-significant. [file 13287_2023_3597_MOESM6_ESM.tiff]

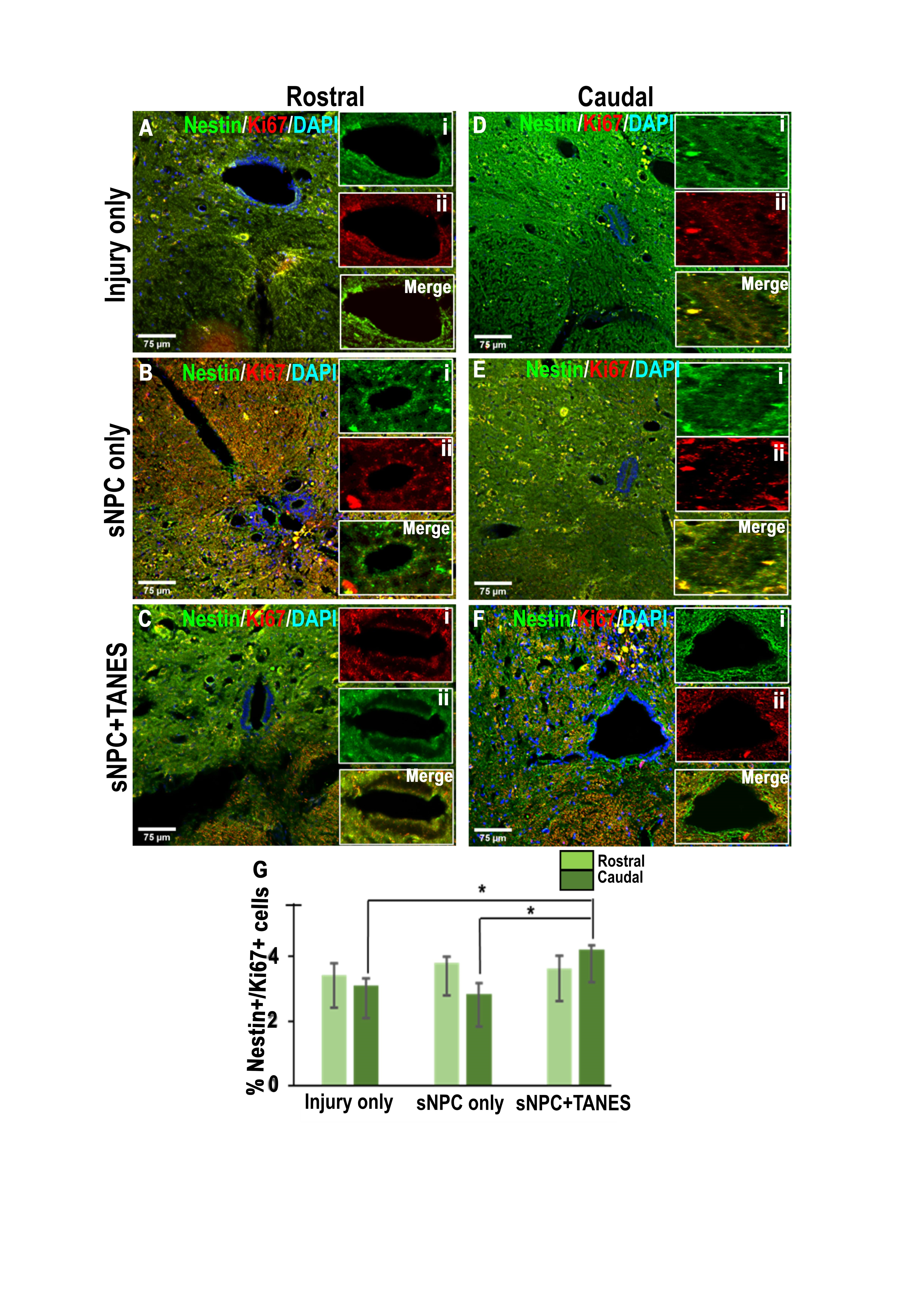

Supplement: Supplementary file 9 — Additional file 9. Supplementary Figure 7. Effect of TANES on the proliferation of endogenous NPCs residing in the central canal rostral and caudal to the lesion site. Spinal cord sections demonstrating merged images of Nestin (green), Ki67 (red) and DAPI (blue) rostral to the lesion cavity (A) Injury only, (B) sNPC only and (C) sNPC+TANES. Caudal to lesion cavity (D) Injury only, (E) sNPC only and (F) sNPC+TANES. Scale bar: 75μm. Higher magnification images are depicted in the boxes of the respective image with (i) Nestin, (ii) Ki67 and (iii) Merged. (G) Percentage of Nestin positive cells co-localized with Ki67 in Injury only, sNPC only or sNPC+TANES groups at 0.6cm rostrl and caudal to the lesion cavity. Data represent mean± standard error of the mean; *p<0.05. [file 13287_2023_3597_MOESM9_ESM.tiff]

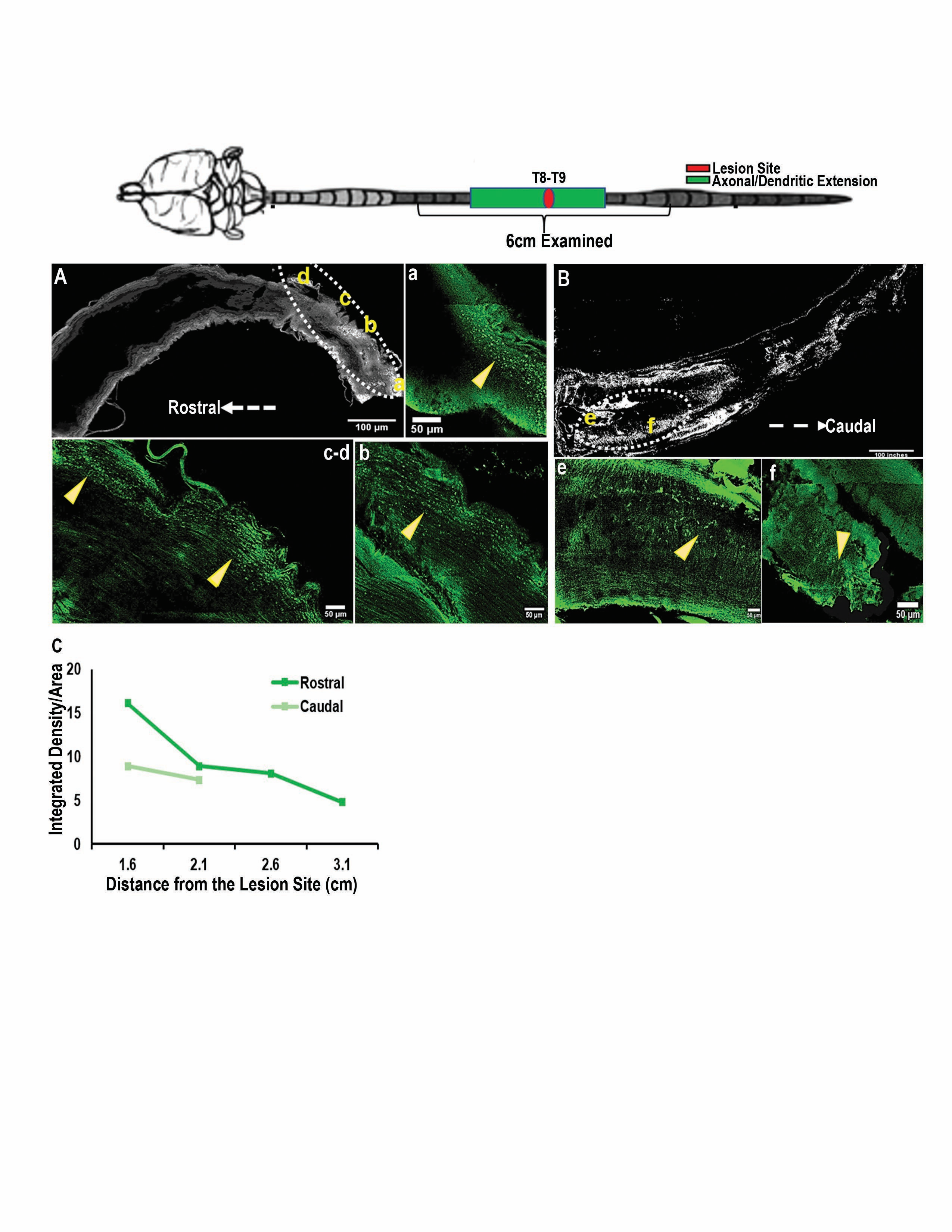

Supplement: Supplementary file 10 — Additional file 10. Supplementary Figure 8. Visualization of axonal/dendritic projections in the spinal cord using tissue clearing. Expression of SC121 represented in green in the whole spinal cord (A) rostral; (a-d) Higher magnification images at 1.6cm-3.1cm rostral to the injury site, (B) Caudal; (e-f) Higher magnification images at 1.6cm-2.1cm caudal to the injury site, 16 weeks after sNPC transplantation and TANES. Scale bar: (A, B) 100μm, (a-f) 50μm. (C) Quantification of relative integrated density per area of SC121 positive projections over the distance. [file 13287_2023_3597_MOESM10_ESM.tiff]
